# Leaving no one behind? Social inclusion of health insurance in low- and middle-income countries: a systematic review

**DOI:** 10.1186/s12939-019-1040-0

**Published:** 2019-08-28

**Authors:** Suzanne G. M. van Hees, Timothy O’Fallon, Miranda Hofker, Marleen Dekker, Sarah Polack, Lena Morgon Banks, Ernst J. A. M. Spaan

**Affiliations:** 1 Radboud Institute for Health Sciences (RIHS), Department for Health Evidence, Radboud University Medical Centre, Nijmegen, The Netherlands; 2 Department of Work and Health, HAN University of Applied Sciences, Kapittelweg 33, P.O. Box 6960, 6503GL Nijmegen, Netherlands; 3 International Centre for Evidence in Disability, London School of Hygiene and Tropical Medicine, London, UK; 4 VU University Amsterdam, Amsterdam, The Netherlands; 5 African Studies Center, Leiden University, Leiden, The Netherlands

**Keywords:** Health insurance schemes, Social inclusion, Vulnerable groups, Universal health coverage, Equity

## Abstract

**Background:**

One way to achieve universal health coverage (UHC) in low- and middle-income countries (LMIC) is the implementation of health insurance schemes. A robust and up to date overview of empirical evidence assessing and substantiating health equity impact of health insurance schemes among specific vulnerable populations in LMICs beyond the more common parameters, such as income level, is lacking. We fill this gap by conducting a systematic review of how social inclusion affects access to equitable health financing arrangements in LMIC.

**Methods:**

We searched 11 databases to identify peer-reviewed studies published in English between January 1995 and January 2018 that addressed the enrolment and impact of health insurance in LMIC for the following vulnerable groups: female-headed households, children with special needs, older adults, youth, ethnic minorities, migrants, and those with a disability or chronic illness. We assessed health insurance enrolment patterns of these population groups and its impact on health care utilization, financial protection, health outcomes and quality of care.

**Results:**

The comprehensive database search resulted in 44 studies, in which chronically ill were mostly reported (67%), followed by older adults (33%). Scarce and inconsistent evidence is available for individuals with disabilities, female-headed households, ethnic minorities and displaced populations, and no studies were yielded reporting on youth or children with special needs. Enrolment rates seemed higher among chronically ill and mixed or insufficient results are observed for the other groups. Most studies reporting on health care utilization found an increase in health care utilization for insured individuals with a disability or chronic illness and older adults. In general, health insurance schemes seemed to prevent catastrophic health expenditures to a certain extent. However, reimbursements rates were very low and vulnerable individuals had increased out of pocket payments.

**Conclusion:**

Despite a sizeable literature published on health insurance, there is a dearth of good quality evidence, especially on equity and the inclusion of specific vulnerable groups in LMIC. Evidence should be strengthened within health care reform to achieve UHC, by redefining and assessing vulnerability as a multidimensional process and the investigation of mechanisms that are more context specific.

**Electronic supplementary material:**

The online version of this article (10.1186/s12939-019-1040-0) contains supplementary material, which is available to authorized users.

## Background

Universal health coverage (UHC) is a key concern in global health policy, especially in low- and middle-income countries (LMIC) [1, 2]. UHC is grounded in the sustainable development goals (SDGs), which aim to ensure healthy lives and promote well-being for all at all ages by 2030, including financial risk protection and equal access to quality essential health-care services [3, 4]. The World Health Organization (WHO) and other international actors consider the implementation and expansion of health insurance central to achieving UHC. In LMICs, different types of insurance have been implemented, which vary in their scale, providers (government vs private sector), and, often, types of beneficiaries [5]. Across most scheme types, existing evidence underscores the importance of health insurance as a tool to enhance UHC [6–11]. For example, in a systematic review on the impact of health insurance in Asia and Africa, enrolment had a positive impact on reducing out-of-pocket spending, while also increasing utilization of health services [7].

However, while evidence suggests health insurance schemes can improve health care utilization and financial protection for its members, they can also risk compromising equity by excluding high-risk and/or vulnerable individuals in society [12]. For example, people living in poverty may not be covered in health insurance if they cannot afford contributions or are not exempted to pay, leading to inequity in enrollment among the most vulnerable in society [13]. Similarly, certain groups, such as older adults, people with chronic illness and people with disabilities are less likely to participate in social protection programs or may have health service needs that are not covered in standard benefit packages [14–19]. While studies on social inclusion and exclusion in LMIC and health are available, there are hardly any systematic studies of how social exclusion may affect access to equitable health financing arrangements [20]. Conversely, studies on health insurance schemes are available, however those do not evaluate social inclusion of specific vulnerable groups as such [7, 21]. In most cases, neither schemes nor governments have rigorously analyzed and aligned enrolment patterns, needed services and benefit packages based on needs of their population [22–24]. If health insurance schemes are truly to be used as a tool to UHC and thus “leave no one behind”, there is a need to evaluate insurance enrolment and the impacts of participation amongst groups that are most vulnerable to exclusion [6, 25].

The WHO Social Exclusion Knowledge Network (SEKN) developed a Social, Political, Economic and Cultural (SPEC) conceptual model, explaining social exclusion not as a ´state´ but as a process, operating along several dimensions and at different levels from the individual to regional and global levels ‘state’ [26]. These exclusionary processes create a continuum of inclusion/exclusion characterized by an unjust distribution of resources and unequal access and return to the capabilities and rights required to enable participatory and cohesive social systems [26]. Moreover, having a particular disadvantage does not indicate that an individual is socially excluded. Rather, it indicates that the individual is vulnerable to social exclusion [17]. Due to inconsistencies in how social inclusion and exclusion are defined and measured, there are no single sets of indicators which for assessing social exclusion as a process [26]; therefore, we searched for a model to identify social determinants for inclusive health systems that provides an approach towards individuals who might be vulnerable to social exclusion. The EquiFrame offers a social determinants approach to assess the extent to which a given policy is consistent with promoting social inclusion, service coverage and reduce barriers to access – all key components of inclusive health systems [27, 28]. In accordance with the WHO, the EquiFrame has given priority to 12 vulnerable groups [29, 30]. This review selected eight priority vulnerable groups that seem most severely affected by exclusionary processes and for whom strategies to expand or improve health insurance plans have been described in general terms only [31]. Those eight groups are female-headed households, children with special needs, older adults, ethnic minorities, displaced populations, chronically ill and individuals with disabilities. The remaining four groups (those with limited resources, increased relative risk for morbidity, mother-child mortality and those living away from services) were based on parameters that have been frequently used in health insurance evaluations (such as income level, urbanization level), or on maternal health and specific disease programs for illnesses with increase relative risk, which have shown to be covered by health insurance initiatives in previous studies [7, 10, 11, 25, 32–38].

Our systematic review evaluates three types of health insurances, on various outcome indicators that evaluate the impact of the particular health insurance scheme. Various types of health insurance are available in LMIC and have different impact on the population they serve. Social health insurance (SHI) are schemes based on mandatory enrolment, often scaled to the national level and provided by governments; SHI involves the formal sector, with payroll taxes for mobilizing funds and pooling risks. Private health insurance (PHI) and community-based health insurance (CBHI), including micro health insurance, involve voluntary enrolment [5, 7]. CBHI differs from PHI by targeting specific population groups, including vulnerable low-income groups [38]. Following the framework of Preker and Carrin [39] and the SPEC model [40], outcome indicators of health insurance schemes included in this review are enrollment rates in schemes and its impact on health care utilization, healthcare expenditures and financial protection, health outcomes and quality of care. As each outcome indicator represents a step followed by the enrollee through the scheme, social exclusion can occur at each of those outcome indicators. Therefore, we assess the way vulnerable groups behave with regard to each reported outcome at group level, in comparison with other (vulnerable) groups or the general population.

To the best of our knowledge, a robust and up to date overview of empirical evidence substantiating and assessing enrollment in and impact of health insurance schemes on health equity among the selected vulnerable groups is lacking [7, 24, 41]. This systematic review aims to address this gap by assessing health insurance enrolment patterns and the impact of health insurance (health care utilization, financial protection, health outcomes and quality of care) in LMIC for the most vulnerable groups – namely, female-headed households, children with special needs, older adults, youth, ethnic minorities, migrants, and those with a disability or chronic illness.

## Methods

### Search strategy

We conducted a systematic search of the literature, adhering to the PRISMA guidelines for systematic reviews [42]. A total of 11 electronic databases were searched (Pubmed, Medline Ovid, Cochrane, Cinahl, Africabib, JSTOR, EconLIT, Scopus, WorldCat, Web of Science, IBSS) for peer-reviewed studies describing access to and impact of health insurance for the defined vulnerable groups in LMIC. Search terms for LMICs, health insurance and vulnerable groups were defined using MeSH and terms from other systematic review on similar topics [7, 10, 43, 44]. See Additional file 1 for sample search string. All searches were performed in December 2017 and January 2018.

### Inclusion and exclusion criteria

Studies were included if they:

- were articles in peer-reviewed journals, reporting randomized controlled trials (RCTs), before-after study (quasi-experimental), interrupted time series (quasi-experimental), cohort, case-control or cross-sectional studies, or qualitative descriptive case studies,
- studied at least one of the three main types of health insurance that are common across LMIC, SHI, PHI or CBHI or ‘mixed’, in which the study does not explicitly state the type of health insurance by reporting only a binary outcome of insured/non-insured, or in which the study evaluates several insurance types.
- studied at least one of the eight vulnerable groups (refer to Table 1) [27]. Studies that reported more than one vulnerable group, were considered in the analysis of each of those vulnerable groups.
- evaluated at least one of the selected outcome indicators of health insurance (enrolment and indicators of impact: health care utilization, financial protection, health outcomes and quality of care) for the aforementioned vulnerable groups, as defined in Table 2.
- were carried out in a country that was classified as a low- or lower-middle or upper-middle income country in either 1995 or in 2017 by the World Bank. The definition of low- and middle income countries is based on the World Bank classification as a low- or lower-middle or upper-middle country in either 1995, or in 2017, to allow for changes in countries’ income status over time [45].
- were published in English.

**Table 1:** Defined vulnerable groups targeted in this systematic review

Female-headed household	Households headed by a woman (including temporarily female-headed households).
Children with special needs	Children with long-term physical, sensory, intellectual or mental health conditions.
Older adults	Referring to older adults.
Youth	Referring to younger age without identifying gender.
Ethnic minorities	Non-majority groups in terms of culture, race, or ethnic identity.
Displaced populations	People who, because of civil unrest or unsustainable livelihoods, have been displaced from their previous residence.
Chronically ill	People who have an illness requiring continuous care.
Individuals with disabilities	Adults with long-term physical, sensory, intellectual or mental health conditions.

**Table 2:** Outcome indicators, definitions, measures and examples of data synthesis

Outcome indicator	Definition and included measures to represent outcome indicator [39, 40]	Examples of synthesis on social inclusion
Enrolment	Actual scheme enrolment, retention or dropout of health insurance measured by rates.	Higher enrolment rates were graded as having a positive effect compared to the general population, since it is assumed to improve access to health services and reduced outlays for health care. If enrolment was higher compared to general population but the difference was not statistically significant (i.e. *p* > 0.05), this was categorized as a positive effect without statistical significance (noted as +^).
Utilization of healthcare services	Defined as utilization of specific healthcare services by the particular populations. Measures include (probability of) visits to health care providers in general during a specified period prior to survey across members and non-members (one year, 6 months), and use of in-patient care or out-patient care or a comparison between those. Utilization was either expressed as percentages or as probability to make use of health care (odd ratios).	Higher probability to receive hypertension treatment, compared to non-member, showed a positive effect (+), equal use of primary care service for last 6 months compared to other groups/general population had no effect (0), for female headed households less outpatient and inpatient visits compared to male headed households showed a negative effect (−)
Financial protection	Defined as protection against catastrophic health expenditures; measured by out-of-pocket expenditures for health care, in absolute terms or expressed as a proportion of total income or total medical expenditure, or measures related to catastrophic health expenditures (absolute or relative) and the net benefits (financial reimbursement) received by scheme members.	Lower catastrophic health expenditures showed a positive effect for the particular group (+), more use of savings or borrowed money for one type of insurance compared to other types was reported as negative effect (−), no effect of a scheme on reducing enrollees’ total medical expenditure was reported as (0).
Health outcomes	Defined as relevant health outcomes for the vulnerable group, e.g. mortality rates, self-assessed general health status, functional limitations.	Reduced mortality rates among people with chronic illnesses was reported as a positive effect.
Quality of care	Defined as the performance of health services in terms quality of health care, e.g. services covered, efficiency of services or trust.	Improved access to medicine was reported as a positive effect (+), low confidence in scheme by the particular group showed a negative effect (−)

Studies were excluded if they:

- were policy reviews, opinion pieces, editorials, commentaries or conference abstract or systematic literature reviews or grey literature,
- were published before 1995, since other reviews on health insurance found few studies before then [7, 46],
- discussed a health financing system other than health insurance schemes,
- did not have a comparison group (e.g. general population, non-vulnerable groups, non-insured), which was necessary to explore equity in enrolment and impact.

### Study selection

We removed duplicate references in Endnote and used the software of Covidence to import all references. Two authors (TO and SH) independently reviewed all references and identified articles that met the inclusion criteria by title and abstract review. The same authors independently read the full texts and decided whether articles should be included for data extraction. Any differences in opinion among the two authors were discussed until consensus was reached and if necessary, a third reviewer was consulted.

### Quality assessment

We assessed the risk of bias of the studies using the Critical Appraisal Skills Programme (CASP) lists [47]. This is a coherent set of checklists suitable to examine the methodological quality of studies with various designs. Two authors (MH and SH) assessed each selected study that passed full-text review independently, using the relevant list per design (qualitative, case-control, cohort, RCT). A combination of the relevant checklists was used assessing the quality of mixed-methods studies. To assess quantitative descriptive studies, like cross-sectional studies, the case-control checklist was adjusted by removing one question about the controls and adjusting the total score with one point. This enabled the reviewers to also assess the quality of quantitative observational studies without case-control or cohort design. We resolved any disagreement by discussion. Percentage scores were calculated as the sum of item scores divided by the total number of relevant items. Studies were categorized as low quality (≤65%), medium quality (observational studies, cross sectional, cohort or qualitative studies scoring > 65% at CASP), high quality (RCT or quasi-experimental studies scoring > 65% at CASP). Studies of sufficient methodological quality (i.e. with a CASP score > 65%) were included for further analysis [48, 49].

### Data extraction

Two authors (MH and SH) extracted relevant findings from all studies independently. Each reviewer used a data collection form to extract the relevant information. Data extracted from final sample of articles included the following:

- Study design
- Setting (country, country income level, recruitment and characteristics of sample)
- Type of health insurance scheme (SHI, PHI, CBHI, mixed)
- Vulnerable group(s) (definition used and means of identification)
- Comparison group
- Reported health insurance indicators grouped into enrolment, utilization, financial protection, health outcomes and quality of care and if reported enabling factors and barriers per health insurance indicator.

This review incorporates SPEC conceptual model by assessing a positive or negative effect on the above outcome indicators of health insurance at group level, compared to other groups or the general population [26]. Reviewers graded each outcome indicator according to the following categories: positive effect (+); no effect (0); negative effect (−); or not assessed, to detect (un) just distributions within the particular outcome indicator or an (un) equal access to the particular services, which may reflect a failure to respond to equity concerns (deliberately, unwillingly or inadvertently) or problems with implementation (refer to Table 2) [28]. Any disagreement in extracted results was resolved through discussion and if necessary (9 articles), by consulting a third reviewer (ES), until consensus was reached.

### Data synthesis and presentation

Given the heterogeneity in study designs, settings, population groups and outcomes, meta-analyses were not possible. Instead, we synthesized the findings descriptively. We descriptively present the main findings regarding each vulnerable group describing the observations for each group per scheme (refer to Additional file 2). If the study reported separate outcomes for several schemes within one type of health insurance, each scheme is presented as one observation. If the study reported about several vulnerable groups, each vulnerable group was assessed independently and presented separately. For each vulnerable group and each type of health insurance (SHI, PHI, CBHI, mixed), we summed the number of observations with a positive effect (+), no effect (0) or negative effect (−) for each outcome indicator (enrollment, utilization, financial protection, health outcomes and quality of care).

## Results

### Study selection

**Fig. 1:**
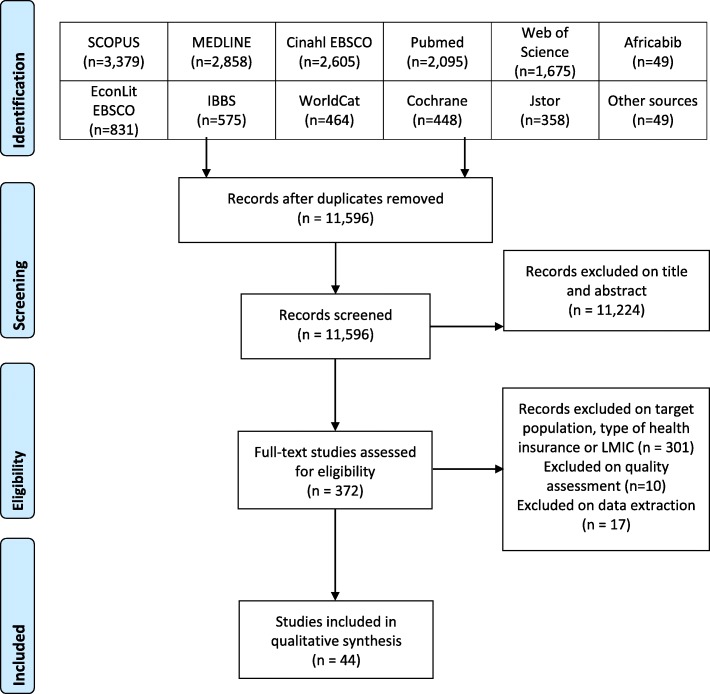
Prisma flow chart

Figure 1 shows the flow chart summarizing the process of study selection. Of the 15,386 citations identified (11,596 after duplicates removed), 11,224 were excluded on title and abstract; subsequently 372 full text studies were assessed of which 44 articles met the eligibility criteria. The number of retrieved citations for each of the 11 databases is presented in Fig. 1.

### Study characteristics

Table 3 provides an overview of the characteristics of the 44 included studies with detailed results per scheme in Additional file 2. The SHI scheme was the most commonly reported type of health insurance (*n* = 28, 55%), followed by mixed schemes (*n* = 10, 20%), CBHI (*n* = 9, 18%) and PHI (*n* = 4, 8%), respectively. The majority (*n* = 41, 80%) of articles reported quantitative observational studies, seven articles (14%) described quasi-experimental studies and three (6%) articles conducted qualitative studies. No RCTs were identified. Studies were carried out in 22 countries across three continents (Africa, Asia and South America) and one study included data from 48 LMICs [50]. This study did not provide specific information regarding the type of insurance and results per country and was therefore not differently weighed to other studies. However, due to large scale of the study in terms of settings and numbers of participants (regarding chronically ill), the study was specifically mentioned in the disaggregation of results. Studies conducted in Asia dominated (*n* = 25, 49%), with a smaller number from Africa (*n* = 12, 24%) and South-America (*n* = 11, 22%). Reported studies on SHI were mainly from Asia, especially China, versus CBHI studies that were conducted mostly in Africa. Most studies (*n* = 40, 91%) were conducted from 2010 onwards, with 24 (55%) observations from 2015 to 2017. No studies were included from before 2005. Target populations reported per scheme were chronically ill (*n* = 34, 67%, out of 51), older adults (*n* = 17, 33%), individuals with disabilities (*n* = 9, 18%), female-headed households (*n* = 6, 12%), ethnic minorities (n = 3, 6%) and displaced populations (*n* = 2, 4%). No articles were found which reported on the enrolment or impact of health insurance for youth and children with special needs.

**Table 3:** Characteristics of studies that met inclusion criteria, observations

Type of scheme*	No of obser-vations**	Vulnerable groups reported***						Quality			Study design****			Data about impact				
		Female headed households	Older adults	Ethnic minorities	Displaced populations	Chronically ill	Disabled	Low	Medium	High	Exp	Obs	Qual	Enrolment	Utilization	Financial protection	Health outcomes	Quality of care
SHI																		
Africa	4		4			2		2	4			4		4	1	1		1
Asia	18		3	2	2	11	2	8	16	2	2	15	2	8	8	12	2	1
South America	6		1			5	2		4			4		4	6	3		
Total	28																	
PHI																		
Africa	1						1		1			1			1			
Asia	0																	
South America	3		1			2			3			3		1	3		1	1
Total	4																	
CBHI																		
Africa	6	4	3			3	2		6			5	1	6	3			
Asia	3			1		3	1		1	2	3			3	2	1		
South America	0																	
Total	9																	
Mixed																		
Africa	1	1				1			1			1		1				
Asia	4	1	2			2	1		3	1	1	3		4	1	1		
South America	2		2			2			2			2		1	2			
Multi continent	3		1			3			2	1	1	2		1	1	2		1
Total	10																	
Total	51	6	17	3	2	34	9	10	44	7	7	41	3	33	28	20	3	4

*SHI Social health insurance, PHI Private Health Insurance, CBHI Community Based Health Insurance

**One study can report about more than one scheme, total studies included is 44

***One study can report about more than one vulnerable group. No studies were retrieved and selected that provide data regarding youth and children with special needs, the 7th and 8th group

****Exp (quasi) experimental design, Obs observational study, Qual qualitative design. No randomized controlled trial was included

Most articles assessed enrolment (*n* = 33, 38%, out of 88), followed by health care utilization (*n* = 28, 32%) and financial protection (*n* = 20, 23%). Within CBHI, articles included data on enrolment and health care utilization relatively more often, while within SHI schemes financial protection was more commonly reported. Studies on PHI schemes did not report on financial protection. Overall, few studies reported on the indicators of health outcomes and quality of care.

### Quality assessment

We excluded ten studies from this review as they were deemed to be of insufficient quality. Incomplete or inadequate reports of measures to define the population (vulnerable groups) or outcomes were major sources of bias in excluded studies, see additional file 3 for study characteristics and the reported bias per study. The vulnerable groups covered in the excluded studies seemed to overlap with the included studies. The number of qualitative studies that was assessed as insufficient of quality is higher than the quantitative studies, compared to the included studies. Sources of bias in the included studies were adverse selection (especially for the chronically ill), lack of adequate adjustment for confounding factors, such as type of chronic disease or distance to health facility, high subject dropouts, recall bias for health care costs, lacking information of definition or measures to identify vulnerable group and non-population-based samples. See additional file 4 for the assessment of each included study, based on the CASP forms.

### Descriptive synthesis of evidence

#### Disaggregation by vulnerable groups

##### Chronically ill

A total of 34 (67% out of 51) schemes in 29 studies assessed inclusion in health insurance amongst people with chronic illnesses. Of these, 18 (53%, out of 34 schemes) focused on SHI, two (6%) on PHI, six (18%) on CBHI and eight (24%) on mixed schemes. 16 (47%) studies were conducted in Asia, six (18%) in Africa, nine (26%) in South America and three (9%) in more than one continent. People with chronic illnesses were mostly defined by long-term conditions or suffering from symptoms more than 30 days.

Most studies reported on the chronically ill and their enrolment rates compared to the general population (refer to Table 4). A positive effect, meaning chronically ill enrolled more than the other group(s) in the study or general population, was reported by six [17, 51–54] out of 11 SHI schemes [17, 51–57]. For CBHI schemes, two [58, 59] out of five schemes [58–62] reported a positive effect, including one high-quality study. For studies reporting of various schemes three [50, 63, 64] out of five schemes [50, 63–66] were positive. Of studies reporting on various schemes, one study by El-Sayed et al. (2015) was graded as high-quality due to a quasi-experimental design in 48 countries [50], highlighting strong evidence for a higher enrollment rate for chronically ill compared to the general population in various health insurance schemes in many LMIC. Another study in Kenya showed that chronically ill had, despite having a borderline significance, 22% greater odds of coverage compared to those without a chronic disease [63]. The proportion of studies with a positive effect on enrollment for chronically ill was 55% for SHI, 40% for CBHI and 60% for studies with mixed schemes. A negative effect of enrolment for chronically ill was reported by three SHI schemes (27%) (two from China and one from Vietnam) [55, 57], one study reporting a CBHI scheme [62] and one with mixed schemes (20%) [65]. Overall, approximately half of the studies found the chronically ill to be more likely to enroll in health insurance than the general population. Studies in Ghana, Senegal, China and India reported a higher prevalence of chronically ill among the insured, pointing to adverse selection [17, 53, 54, 67].

**Table 4:**
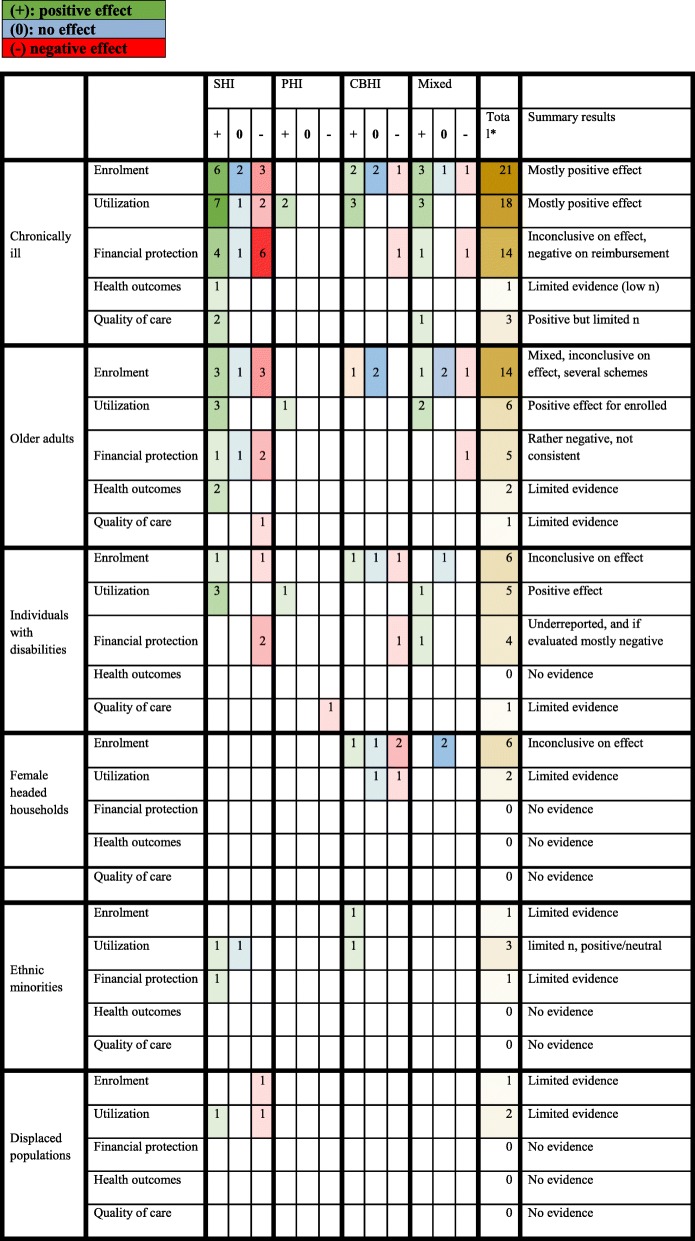
Effect of types of health insurance on different outcomes per vulnerable group

For health service utilization, seven observations (67%) of SHI schemes [51–53, 56, 68, 69] reported a positive effect out of nine observations [51–54, 56, 68, 69]. One quasi-experimental study of individuals with hypertension demonstrated that those with health insurance had a 29% higher chance to receive hypertension treatment compared to subjects with normal blood pressure [68]. Studies of each of the other health insurance scheme types also reported only positive effects, including the high-quality study by El-Sayed et al. (2015) [50, 59, 64, 69, 70]. Those studies provide evidence of a link between having health insurance and utilization of health care when suffering from chronical illness.

In terms of financial protection, some results were positive (*n* = 4, 36%) (but often not significant) [50, 54, 71, 72], however, most were negative (*n* = 6, 55%) [51, 55–57, 67, 73] for SHI schemes, of which most studies were conducted in China. One SHI scheme (9%) in Indonesia (Jamkesmas insurance) showed no effect in financial protection in a way that chronically ill were not being required to pay for additional medical expenses, as much as other patients who faced hospitalization [73]. Two studies with mixed schemes reported on financial protection, including one high-quality study by El-Sayed et al. (2015), reporting a positive effect by decreased likelihoods of borrowing or selling assets to pay for health services [50]. The other mixed scheme study reported a negative effect on financial protection whereby insured individuals aged 50+ suffering from chronic illness had higher CHE during the previous year compared to non-insured [74]. Only one study regarding a CBHI scheme reported on financial protection and found a negative effect for chronically ill [62]. In summary, we found that health insurance schemes could prevent CHE; however, chronically ill experienced insufficient financial protection and reimbursements rates for both SHI and CBHI were generally very low.

The other categories of outcome indicators, health outcomes and quality of care, were reported less frequently. One study on health outcomes, showed a positive effect by reduced mortality rates among people with chronic illnesses [69]. For quality of care, three studies reported positive effects for chronically ill, one on access to medicines in five LMIC [75], one on higher user satisfaction in Chile [56] and one regarding increased awareness on high blood pressure [68].

To summarize, studies showed reasonably strong evidence for higher enrollment rates for chronically ill in (various) health insurance schemes compared to the general population, and once insured, chronically ill seem to utilize health services. There was some evidence that health insurance schemes may prevent CHE, however reimbursement rates still seemed low.

##### Older adults

The second most frequently reported group was older adults; 17 (33%) studies assessed inclusion in health insurance amongst older adults. Of these, eight (47%) focused on SHI, one (6%) on PHI, three (85%) on CBHI and five (29%) on mixed schemes. Five (29%) studies were conducted in Asia, seven (41%) in Africa, four (24%) in South America and one in more than one continent. Older adults were mostly defined as individuals being 50 years and above or 60 years and above.

Findings in terms of enrolment are inconsistent; the SHI schemes found an equal number of observations showing positive effects (*n* = 3, 42%) [76–78], negative effects (n = 3, 42%) [17, 77] and one scheme (14%) with no effect in enrolment (refer to Table 4) [51]. For example, one study from Ghana reporting on the national health insurance program demonstrated a positive effect on enrolment and financial protection for individuals aged 70 and above who are exempted from enrolment fees and a negative effect for the age group 60–69 who are not exempted from enrolment fees, both compared to the enrolment of the general population [77]. Looking at the other schemes, PHI, CBHI and mixed schemes, several studies showed no effect on enrolment among older adults (*n* = 4, 57%) [41, 58, 60, 79]. The exceptions were one study of a PHI scheme which found a negative effect on enrollment among older adults in China [65] and one study of mixed schemes from Mexico which found a positive enrolment effect for older adults [64].

With regards to utilization of health services, all schemes [51, 64, 70, 76, 80, 81] report a positive effect on health care utilization for older adults, except one CBHI scheme [41] describing no effect.

Five health insurance schemes reported on financial protection, four on SHI (80%) [51, 76, 81] and one on mixed schemes (20%) [74]. Only one study reported a positive effect on financial protection, by the aforementioned individuals aged 70 and above in Ghana receiving the exemption fees [76]. Two studies report a negative effect. One study found that among older adults with a chronic illness in six countries from Asia, Africa and South-America, the insured had higher rates of CHE, likely due to frequent visits to health facilities because of increased health needs [74]. Another example from a Mexican study showed that older adults used more savings to access care [51]. One study from China, reporting on the New Cooperative Medical Scheme, showed no effect on reducing enrollees’ total medical expenditure and out of pocket payments [81]. In sum, results showed in a more negative picture of financial protection for older adults accessing health care.

Addressing health outcomes, this study found improvements in enrollees’ activities of daily living and cognitive function but no improvement in self-assessed general health status. Only one other study from China reported on health outcomes for older adults and found a positive effect on health status because of being insured [82]. Assessing quality of care, one negative effect for SHI, (low confidence in scheme) [76] is reported.

In summary, the effect of enrolment rates for older adults compared to the general population seemed mixed, however studies indicate that being insured appeared to have a positive effect on health care utilization. The impact of health insurance on financial protection was rather negative and there is limited evidence on the impact of health insurance on health outcomes and quality of care for older adults.

##### Individuals with disabilities

Addressing individuals with disabilities, 9 (18%) studies assessed inclusion in health insurance. Of these, four (44%) focused on SHI, one (12%) on PHI and four (44%) on CBHI. Four (44%) studies were conducted in Asia, three (33%) in Africa and two (22%) in South America. Individuals with disabilities were mostly defined as people with motor impairments, or with difficulties in certain functional domains or not specified.

Regarding enrollment, studies yielded inconclusive findings, with an equal number of studies finding positive [51, 58], neutral (of which one quasi-experimental study) [61, 83] (enrolling more or less than general population or other groups) and negative [62, 84] effects in enrolment. On utilization of health services, all studies reported a positive effect (100%) [14, 51, 83, 85, 86]. One high-quality study from Vietnam, assessing several types of schemes, reported positively about financial protection [83], demonstrating that insured individuals with disabilities spent 84% less on health care than those uninsured. Three out of four studies [51, 62, 86] reported a negative effect on financial protection. One study from Mexico demonstrated a negative effect of the Seguro Popular health insurance scheme on financial protection in which older adults with disabilities use more savings or borrow money or sell assets to access care compared to individuals with disabilities in the pre-existing social security health insurance [51]. One study about PHI found that individuals with mental illness who have no health insurance pay private service rates but for a slightly lower price compared to those having private health insurance [85]. Another CBHI scheme found that for individuals with physical disabilities, costs for drugs, medical devices and hospital care were not or not fully covered, and it was impossible to get a loan for medical devices [62]. Only one study reported a positive effect on financial protection [83], contradictory to a study from the same country and health system in Vietnam [86].

In summary, the evidence provided inconclusive findings on enrolment and generally positive effects of health insurance on utilization of services for individuals with disabilities. Financial protection was underreported, however the majority of included studies showed negative results. No reports were found on health outcomes and quality of care.

##### Female-headed households

Female-headed households were reported in 6 (12%) studies, of which 4 (67%) focused on CBHI and 2 (33%) on mixed schemes. Five (84%) studies were conducted in Africa and one (17%) in Asia. The studies reporting about female-headed households identified a female headed household by the gender of the household head.

The studies that reported on enrolment of female-headed households in CBHI schemes showed limited and inconsistent results (refer to Table 4), demonstrating no effect (*n* = 3, 50%) [60, 63, 79], a non-significant positive effect (17%) [87] or a negative effect (*n* = 2, 33%) [58, 61]. Two studies reported on utilization of health services, one study showing that gender of the household had no significance in determining use of health care service [61]. The other study demonstrated a negative but insignificant effect, showing female headed households had less outpatient and inpatient visits compared to male headed households [87]. No reports are made about the other impact indicators for this particular group.

Overall, female-headed households were minimally addressed in the literature, and if addressed, it was mainly in relation to CBHI schemes with inconsistent results on enrolment and no information on impact of health insurance.

##### Ethnic minorities, displaced populations, youth and children with special needs

No studies were identified on youth and children with special needs. Ethnic minorities were only reported on in three studies (refer to Tables 3 and 4), only in Asian countries [86, 88, 89]. One community health insurance scheme in India reported a positive impact on enrolment for ethnic minorities and a higher hospital admission rate for insured compared to non-insured [88]. One reason suggested by the authors could be a higher incidence of chronic and major ailment. For health care utilization as well as financial protection, a study from Vietnam showed a positive effect for the insured among ethnic minorities, health insurance increased the likelihood to use inpatient care and community clinic usage, decreased probability of self-treatments and the insured had lower CHE [86]. One study reporting about the national scheme in Thailand reported no association between health insurance and increased health care use for Thai ethnic minorities [89].

The least reported group were displaced populations; health insurance appeared to have negative impact for ethnic minority migrants, on enrolment and utilization in one study in Thailand, compared to Thai citizens [89]. However, another study also in Thailand had a positive (insignificant) effect on utilization of services for displaced populations [90].

In summary, ethnic minorities and displaced populations are minimally reported in health insurance literature and no overall conclusion can be made from the included literature.

#### Disaggregation by type of health insurance scheme

An analysis of social inclusion by type of scheme suggests an observable pattern in terms of reporting on coverage of vulnerable groups. Results on health insurance enrolment of vulnerable groups are most commonly reported in studies on SHI (*n* = 28, 52%). More specifically, this type of scheme focused most commonly on chronically ill (*n* = 18, 64%) or older adults (*n* = 9, 32%) and, to a smaller extent, on individuals with disabilities, especially in South-America and Asia. Although fewer studies of CBHI schemes (n = 9, 18%) were identified compared to SHI schemes, these studies reported the enrolment of a broader range of groups, including chronically ill (*n* = 6, 67%), female-headed households (*n* = 4, 44%), older adults (*n* = 3, 33%) and individuals with disabilities (n = 3, 33%) and reported but scarcely about displaced populations and ethnic minorities. Few studies on vulnerable populations and private health insurance were identified.

#### Disaggregation of reported enabling factors and barriers to health insurance

The studies in this review reported enabling factors and barriers in several studies (*n* = 28), specifically for enrolment (*n* = 19, 68%) [17, 41, 50, 51, 54, 58, 60, 62, 67, 76–81, 84, 87, 88, 91], utilization of health care services [83, 90] (n = 2, 7%), financial protection [71–73] (n = 3, 11%), health outcomes [82] (n = 1, 4%) and quality of care [69, 85] (n = 2, 7%) (refer to Additional file 2, right column). In summary, reported enabling factors on enrolment were: higher household gross income per capita, having formal education or employment, large household size or children under 15 in the household, living near the health facility or in an urban area, having been hospitalized, presence of catastrophic illness, having a less severe disability, high level of understanding of risk pooling and belonging to community groups or the majority religious group. Reported barriers for enrolment were: being poor, having low level of awareness or information about the health insurance scheme, lower political participation, unsafe environment, limited access to information, having reduced cognitive function, inappropriate benefit packages, waiting lists, lack of satisfaction of providers’ behavior and lack of trust in the scheme. Factors such as being male, being older or being married were reported both as enablers and barriers.

With regard to utilization of health care services, enabling factors were having more household members and proximity to the hospital, and barriers were age and low income. For financial protection, a higher income level was an enabling factor (household gross income per capita), for example for chronically ill in China. A barrier was inappropriate benefit packages. For health outcomes, a reported enabling factor was having proper leisure activities, and barriers were being an older woman, being a widow or having a low income. Lastly, for quality of care, the only reported barrier was presence of waiting lists.

## Discussion

This paper provides a comprehensive review of studies that have assessed enrolment and impact of health insurance for specific vulnerable groups. The most notable finding is the dearth of high-quality evidence which evaluates access to and impact of health insurance schemes for vulnerable groups in LMIC, such as individuals with disabilities, female-headed households, displaced populations, ethnic minorities, displaced populations, youth and children with special needs. Despite all attention paid to UHC, this calls for more attention to the issue of equity for specific vulnerable populations in health insurance, in line with the recommendations of the WHO [92]. The existing evidence gathered from this systematic review and its policy implications are discussed below.

### The reported vulnerable groups

This literature review revealed that available knowledge about health insurance inclusiveness is sparse for each of the vulnerable groups and of variable quality. Out of the eight included vulnerable subpopulations, the chronically ill were the most commonly reported, followed by older adults and individuals with disabilities. These subpopulations might have a clear set of ‘conditions’ or ‘non-communicable diseases’ which are relatively easy to define and therefore, easy to tackle within health insurance impact evaluations. This health-related ‘state’ of vulnerability might not be the case for the other, less addressed groups, such as gender, ethnic minority or citizenship (for displaced populations), which are especially influenced by political or cultural dimensions. One exception here is that children with special needs are unreported as subgroup based on our review findings, probably because children are often included in household enrolment evaluations, where the insurance status of the head of the household is taken as an assessment of enrollment. Other reviews on the impact of health insurance schemes for children also did not report findings on children with special needs due to disabilities [33, 37].

### Health insurance indicators addressed: enrollment and impact

Henceforth, we discuss our results by the SPEC-by-step tool, based on the SPEC conceptual model [40]. The first step, “awareness”, ideally targets all people to become aware of health insurance schemes. This systematic review aimed to assess actual enrolment in health insurance, rather than levels of awareness. However, vulnerable groups are more likely to face social exclusion/discrimination and less likely to be included in initiatives that may promote awareness of health insurance (e.g. in identification process, risk pooling, awareness raising activities) [10, 77, 93]. Though we did not specifically assess this step, we believe that with regard to inclusionary processes, equity starts by being reached and empowered in order to decide about the involvement in health insurance schemes.

The next step is “enrolment”, defined as registration to a health insurance scheme. For most of the eight vulnerable subpopulations in this review, enrolment numbers are scarcely reported. For the chronically ill, and to a lesser extent for older adults, there is some limited evidence that they are more likely to enroll than the general population [44, 94]. This phenomenon of adverse selection can be defined as strategic behavior by the more informed people in a contract against the interest of the less informed people such as those in the informal sector [95]. This review shows that older adults and chronically ill, those who seem mostly in need of frequent health care services, tend to enroll more. Also, it is possible that this review included studies where enrolled individuals in households had the worst health status and non-enrolled individuals in household had the best health status, meaning inequity in enrolment among vulnerable groups within households still exists [96, 97]. Another possibility is that adverse selection manifests itself through healthy people choosing managed, well-organized care and less healthy people choosing more generous plans, and adverse selection is therefore more likely to happen in voluntary insurance schemes [98]*.* Ways to tackle adverse selection, and therefore ensure better inclusion of other groups, are mandatory scheme enrolment, enrolment at the household level or introduction of a waiting period [7, 36]. Notwithstanding a possible positive effect on enrolment found in this review through adverse selection, it was reported in previous reviews on individuals with disabilities that access to social protection programs still appears to be far below need [18, 20]. Possibly, enrolment remains low due to high premium rates, hidden costs to enroll, e.g. travelling with assistance, and intensive information interventions or reforms using voluntary contributory mechanisms have no effect on enrolment of the less affluent [13, 99, 100]. Despite the aim of risk pooling [11], the health care delivery system should be equitable and thus favor vulnerable population groups in order to increase their utilization of health care services and reduce as much as possible their exclusion to affordable health care [101].

Being enrolled in a health insurance scheme with a valid membership card should in principle ensure access to the health care benefit package for them. The positive results for the “accessing care” step in this review concur with previous reviews that for the reported groups, health insurance membership can increase utilization of health care services [7, 95].

In terms of the “benefits” step, due to inconsistency in findings, we cannot draw clear conclusions on the impact of health insurance on financial risk protection (e.g. use of savings, CHE), health outcomes (e.g. access to medicines) and quality of care for the eight vulnerable subpopulations. Further, it remains unclear who gets what services and in what proportion health services and related costs are really covered and reimbursed [102].

On financial protection, for instance, our review suggests that for chronically ill, older adults and individuals with disabilities, formal insurance schemes do not guarantee protection from CHE, despite greater needs for health services [50, 81]. Our findings extend those of others, confirming that households with older adults, young children or chronically ill borrow money or sell assets to finance illness, underlining the bilateral link between health and poverty [43, 103]. Looking at a wider spectrum of health financing arrangements, a Nigerian study found that households with older adults participating in informal health financing arrangements (other than health insurance schemes) were less likely to incur CHE than those with formal schemes [104]. Unsurprisingly, there is a discrepancy between what is formally covered and what is in reality paid for, and therefore, it is likely to underestimate the true extent of economic poverty among vulnerable groups [43]. Despite the fact that some of the results do show that health insurance, to a certain extent, serves as a mechanism to protect from CHE, the impact of health insurance on poverty remains insufficiently clear for vulnerable subpopulations [7, 38, 95, 105]. Hence, the notion of equity in utilization and financing of care may not be enough to judge whether a health system protects the income of vulnerable groups against expensive health care use [106]. What is often overlooked is that social protection programs such as health insurance not only exist for poverty reduction but also for poverty prevention, by helping to prevent people to move into the group of the extreme poor [107].

As we turn to the delivery of care, limited reports are available on the received quality of care by the included vulnerable groups, whilst higher quality of care could enhance member renewal decisions [44]. A way to assess quality of care could be through vertical equity, using indicators of access and use of health care according to needs [108]. Lastly, due to methodological challenges, there are inconclusive results on the impact of health insurance on health status. Evidence in our review is insufficient to understand the complex causal chain behind the impacts of UHC programs on health status [11].

### The complexity of health insurance research

Generally, social exclusion is preferably viewed as a process rather than a ‘state’, in the latter case risking to neglect the relational nature of these ‘states’ and the exclusionary processes generating them [26]. Health insurance evaluations often use parameters such as income and household size, rather than parameters that refer to societal risk factors to exclusion. In our review, we found this measurement issue particularly in the low-quality studies, using unclear or inconsistent measures to define the vulnerable group, or in the vulnerable groups that were based on socio-demographic characteristics only (such as older adults, youth, female-headed households), in which no measurements are taken that include the socio-cultural context of the person ‘at risk’ of vulnerability. Therefore, the findings of the review should be interpreted with caution since we recognize that exclusionary or inclusionary processes will impact in different ways to differing degrees on different groups and/or societies at different times.

Few studies in this review qualified as high-quality impact evaluations based on the study design, risk of bias or insufficient information provided. No RCTs or interrupted timeline series studies were identified. High-quality impact evaluations appear difficult to apply to health systems, both for economic reasons (costly and labor intensive) and for ethical and political reasons. Specifically defining what is meant by ‘the poor’ (e.g. the very poor, indigent and vulnerable) and who qualifies to be categorized as such is challenging, costly and politically sensitive [77, 109, 110]. Furthermore, there are ethical issues around withholding of services in order to create control groups [110]. In conclusion, our review shows that there is a need to develop more specific social determinants and equity indicators for specifically defined vulnerable population groups to use for health insurance scheme impact evaluations. We also suggest the usefulness of including the social, political, economic and cultural dimensions for appropriately measuring the population coverage, possibly by using tools such as the SPEC-by-step tool [40].

### Health insurance impact on equity and universal health coverage

Returning to the question of UHC, this can be promoted through actions to improve efficiency, equity in the distribution of resources as well as transparency and accountability [111]. Equity needs to be differentiated from distribution. Financial reforms that improve equity in the distribution of resources can also lead to improvements in equity in the use of services. However, equal access may not be sufficient to improve the situation of vulnerable individuals. The overall aim of UHC is to match and optimize the distribution of resources to the relative health service needs of different individuals and groups in the population [112]. Therefore, on one hand, vulnerable subpopulations may need additional assets to participate in general (redistribution, advancement to at-risk groups) and if this happens, it is important to understand how this ´targeting´ has been realized. On the other hand, for the sustainability of the system, too much favor can be detrimental in economic, social and political terms, so a right balance will have to be found. A new health insurance scheme is either designed for the purpose of making its members better off in terms of health or designed and intended to serve as an agent of change to improve equity in the use of quality health services and its financial protection for the entire population, positioned in a broader context of “leaving no one behind” [111]. For both cases, and for voluntary as well as for mandatory schemes, good coverage for some people comes at the expense of the rest of the population. Therefore, the interests of the schemes can be in conflict with UHC objectives at the level of the entire system.

Because health systems aim to promote universal protection against financial risk, health insurance schemes in low- and middle-income countries undertake various initiatives to reach the vulnerable members of the populations such as discount cards, conditional cash transfer (CCT) programs, exemption fees or free enrolment for vulnerable populations [7, 46]. Exemption fees, such as one study in this review reported in Ghana [76] and cash transfers for vulnerable children and families seem ways to promote universal protection and health coverage by social protection programs [113, 114]. Thus, to do so in social protection programs, how do we get everyone around the table? Study findings show that the limited effectiveness of other programs for cooperation is primarily linked to political factors -such as power relations, interests and incentives of the various actors [115]. In other words, power analyses should be central to inclusive development research and social protection programs such as health insurance [116].

Reflecting on the results of our study and the universality of those results, we compare in brief how social inclusion and equity towards health insurance has been captured in high-income countries. One review evaluating equity within UHC in high-income as well as LMICs found that studies from high-income countries tended to focus on access to specialized services or for specific diseases, and those studies focused on distinct populations such as children, elderly or psychiatric patients, rather than the population as a whole [117]. In our study we found a few studies focused on distinct populations or specific diseases, however most studies focus on the impact of health insurance for the whole population. This could possibly be the case due to the relative new nature of health insurance schemes in LMIC and the limited coverage of services, often emergency care and basic in- and outpatient services versus specialized care in high-income countries. Nevertheless, this same study revealed findings in the research describing inequities in receipt of specialized health care in Canada and Australia in spite of insurance systems [117]. Studies targeting one or more specific vulnerable groups with regard to health insurance describe comparable results to our review also. For example, among chronically ill, two systematic reviews showed that being covered by health insurance improved outcomes on health care utilization and health outcomes [118, 119]. Two studies covering several European countries, found that vulnerable populations, such as poor citizens, elderly citizens or elderly with chronic conditions, had (catastrophic) medical expenses and thus health insurance did not provide adequate financial protection [120, 121]. Furthermore, a review of systematic reviews analyzed to what extent hosting advanced countries provide equal access to health insurance for migrants. As in our systematic review, this study also showed the lack of empirical evidence on enrolment of migrants in health insurance and the need for strategies such as information and application support to expand health insurance coverage in vulnerable populations [122].

On the measurement issue of social inclusion in health systems and health insurance, a study published by WHO on financial protection in Europe, found that the association between gaps in population coverage and financial hardship is weak because people lacking coverage usually only account for a small share of the population, and European countries generally provide all residents with access to emergency services, which is often not the case in LMIC [123]. However, the incidence of catastrophic health spending and financial protection still varies hugely among households in Europe, especially among countries that joined the EU after 30 April 2004 [123, 124]. Similar strategies, such as exemptions for poor people and regular users of health services – e.g. people with chronic conditions, are used in those countries to protect against financial hardship (WHO Europe) [123].

In conclusion, we observe similar achievements and challenges towards equity of health insurance schemes and its impact for particular vulnerable groups between LMIC and high-income countries, however the level of financial protection and access to emergency care of or non-specialized care might be covered more sufficiently in high-income countries.

### Strengths and limitations

To our knowledge, this is the first systematic review of social inclusion of the eight defined vulnerable groups regarding the access and impact of health insurance. A strength of this study was the extensive search in 11 databases covering literature from 1995. As our harvest of papers indicate, the studies on health insurance impact evaluations is on the rise, reflecting a rise in health insurance schemes more recently [7, 125]. With the design of this systematic review, we could not incorporate changes per country per scheme. Also, this review did not integrate the specificity of each country and therefore, no conclusions can be drawn on country-specific levels of equity and its implications for the particular scheme and related policies. Nevertheless, by focusing on specific vulnerable groups related to several types of health insurance instead of a broad categorization of parameters, we were able to collect a relevant data set that could inform policy and research in a way that it provides a picture of the current extent of evidence in the literature regarding inclusion of health insurance schemes for the targeted vulnerable groups. However, since we chose not to include studies about all other possible social determinants or individuals at-risk of vulnerability, such as sex workers, individuals who are homeless or living in institutions, the data about inclusionary processes are incomplete. Also, the definition of the included vulnerable groups was not consistent in each study, for example varying age minimums for older adults, or defining the presence of a chronic illness. Furthermore, we choose a rather generic definition of chronic illness in the search strategy, not defining each chronic condition or specific disease programs, in order to prevent an unmanageable amount of hits that seemed not related to health insurance schemes. As a result, only one study on the impact of health insurance on HIV/AIDS management is included.

Another strength was the comprehensive approach to evaluate health insurance impact allowing a wide variety of study designs and outcome indicators ranging from the inclusiveness of enrolment to the quality of delivered care and degree of financial protection once being insured. However, its assessment of a positive effect, no effect or a negative effect was not always straightforward because control groups were often not clearly described.

Lastly, only English written articles and peer-reviewed articles were included while in many LMIC other official languages or formats such as reports might be used to publish results. The conclusions of this review may be considered general or preliminary by nature, due to the diversity of the included studies and the low levels of evidence found. Nevertheless, in the absence of more precise research findings for most of the vulnerable groups considered here, our results provide a good first indication of the extent to which inclusionary processes towards equity in health insurance schemes in LMIC is represented in the literature.

### Policy implications and future research

The review findings point to a major gap in knowledge regarding the inclusiveness of health insurance schemes and its impact on health outcomes, quality of care and financial protection. What is the magnitude of the gap and what additional measures can bridge this gap? Regarding the first part of this question, it is clear that more data and disaggregated analysis is needed. Social protection programs, including health insurance schemes, can help build human capacity and enhance the stability of economic growth [116]. As we found, there is a need to assess the impact on the utilization of care, its quality, and the effects on invisible CHE and health outcomes for those who are prone to increased utilization of health services. Results of this review point to the need of country specific policies and impact evaluations for vulnerable groups to be used to improve the inclusion of and benefits for those groups in health systems in particular health insurance schemes. This could be assessed by evaluating the cost of health insurance and medical benefits, the level of financial protection, e.g. reimbursement rates, and whether health insurance schemes satisfy the needs of specific subpopulations. For other groups, we have seen that community-based mechanisms are highly useful [13]. Community-based mechanisms seem to recognize the diverse needs of the vulnerable subpopulations though how it contributes to access to health care is not shown on a large scale. This is not a short-term remedy and long-term commitment of governments is required in order to lead to equal health outcomes. A strategy to achieve UHC is through a risk-adjusted equalization of budgets to health care providers or purchasing agencies; this may improve equity in the distribution of resources and services and the reduction of fragmentation in pooling to enable greater financial protection and equity in the distribution of resources and services [111].

We then address the issue of additional measures to bridge the gap in knowledge in the inclusionary process of health insurance. Systematic health systems research needs to be strengthened by assessing how criteria for prioritizing groups that are disproportionately affected change, as well as equity impact of cost sharing, especially for the most vulnerable [11]. Accordingly, the issue of targeting needs must be critically examined. Possible solutions to improve quality of research are to combine quantitative analysis of effect with qualitative information describing context and implementation issues and to seek for measures that assess equity and the effect of changes in policies [95]. Future evaluations should consider mixed or qualitative approaches such as realist evaluation that seek to answer the questions of what works for whom in which circumstances in order to bring about various mechanisms in the contexts in which they are delivered [126], such as one realist review by Robert et al. (2017) resulting in key mechanism to seek free public healthcare in sub-Saharan Africa [127]. In this approach, both qualitative and quantitative methods can be used to trace these mechanisms.

## Conclusion

This review provides an assessment of the available evidence on the impact heterogeneity of health insurance schemes in LMIC by means of a systematic review of the literature. Despite a sizeable amount of literature published on health insurance there is still a dearth of good quality evidence, assessing equity and inclusion of specific vulnerable groups. People with disabilities and individuals who belong to female-headed households, youth, children with special needs, displaced populations and ethnic minorities were minimally reported within health insurance impact evaluations. Based on the evidence available from the studies that assess equity, social exclusion is visible in both the access to health insurance schemes and in lower financial protection. From a social inclusion viewpoint, health insurance has not yet shown to serve as an optimal tool to UHC, in a way that vulnerable groups are covered, from being aware and enrolled in health insurance schemes to proven impact on financial protection and improved health outcomes once carrying a health insurance card. This review also clearly demonstrates that current literature - by using common parameters such as income - is insufficiently clear on the impact of HI on specific vulnerable groups in terms of social inclusion. We therefore propose to move beyond a focus on the overall group of the poor, to develop specific measures for those at risk of exclusion and to assess inclusionary processes. With such measures, more sounder conclusions can be drawn on the social inclusion impact of health insurance in LMICs.

Furthermore, the review shows that while some groups are underreported, there is moderate evidence that mostly SHI schemes do enroll the chronically ill, implying a positive effect on social inclusion. CBHI schemes show some studies addressing various vulnerable groups. For chronically ill, older adults and individuals with disabilities we found that once being enrolled in a scheme, utilization of health care services seems to increase. Regarding financial protection among several schemes dealt with here, the picture shows negative or insufficient effects for chronically ill, older adults and individuals with disabilities, and incomplete results for other underreported groups. Minimal evidence was found that being a member of a health insurance scheme could prevent from CHE and reimbursement rates and ‘real’ (including indirect) costs could be easily underreported. No conclusions can be drawn for the included vulnerable groups on quality of care and health outcomes.

Evidence should be strengthened within health care restructuring systems to achieve UHC by redefining the dimensions to identify vulnerability and the investigation of in- or exclusionary mechanisms that are more context specific. We recognize that for health insurance schemes, different strategies are required to include individuals who are ‘at risk’ of vulnerability and to assess exclusionary processes in order to “leave no one behind”.

## Additional files

Additional file 1: Search strategy Pubmed. Example search strategy. (DOCX 18 kb)

Additional file 2: Included studies. Summary findings from included studies. (DOCX 147 kb)

Additional file 3: Low quality studies. Low quality studies. (DOCX 37 kb)

Additional file 4: Quality assessment. Quality assessment based on forms from CASP. (DOCX 73 kb)

## Data Availability

Not applicable (systematic review using data published in primary studies).

## Abbreviations

CASP: Critical Appraisal Skills Programme
CBHI: community-based health insurance
CHE: catastrophic health expenditure
LMIC: low- and middle-income countries
PHI: private health insurance
RCTs: randomized controlled trials
SDGs: sustainable development goals
SEKN: Social Exclusion Knowledge Network
SHI: Social health insurance
SPEC: Social, Political, Economic and Cultural
UHC: Universal health coverage
WHO: World Health Organization
