# Supplementary material for: Leaving no one behind? Social inclusion of health insurance in low- and middle-income countries: a systematic review
Source: Int J Equity Health. 2019 Aug 28;18:134. doi: 10.1186/s12939-019-1040-0 (PMC6714392; doi:10.1186/s12939-019-1040-0)
Supplement: Supplementary file 1 — Search strategy Pubmed. Example search strategy. (DOCX 18 kb) [file 12939_2019_1040_MOESM1_ESM.docx]

Additional file 1.

Search strategy Pubmed

**Pubmed – searched 16/12/2017**

Search by using following instruction

Use an asterisk (*) for **truncation**.

PubMed uses the **MeSH thesaurus**.

Put quote marks (" ") around **phrases**.

You can use **brackets**to ensure the logic is carried out in the correct order eg health and (promotion or education)

[MH] = MeSH thesaurus

[TIAB] = title and abstract

| *Low and Middle Income Countries* | | # Hits |
| --- | --- | --- |
| 1 | developing countr*[MH] OR developing countr*[TIAB] OR developing nation*[TIAB] or developing world[TIAB] | 118,112 |
| 2 | least developed countr*[TIAB] OR least developed nation*[TIAB] OR least developed world[TIAB] OR least-developed countr*[TIAB] OR least-developed nation*[TIAB] OR less-developed countr* OR less-developed nation*[TIAB] OR less developed countr*[TIAB] OR less developed nation*[TIAB] OR under-developed countr*[TIAB] | 1,763 |
| 3 | under-developed countr*[TIAB] OR under developed countr*[TIAB] OR underdeveloped countr*[TIAB] OR under-developed nation*[TIAB] OR under developed nation*[TIAB] OR underdeveloped nation*[TIAB] OR under-developed world[TIAB] OR under developed world[TIAB] OR underdeveloped world[TIAB] OR under-developed econom*[TIAB] OR under developed econom*[TIAB] OR underdeveloped econom*[TIAB] | 1,117 |
| 4 | third world countr*[TIAB] OR third world nation*[TIAB] OR third-world countr*[TIAB] OR third-world nation*[TIAB] | 1,032 |
| 5 | Low income population[MH] OR Low income population[TIAB] OR low and middle-income countr*[TIAB] OR low and middle income countr*[TIAB] OR low and middle-income nation*[TIAB] OR low and middle income nation*[TIAB] OR low- and middle-income world[TIAB] OR low and middle income world[TIAB] OR low and middle-income econom*[TIAB] OR low and middle income econom*[TIAB] OR low income countr*[TIAB] OR middle income countr*[TIAB] OR low-income countr*[TIAB] OR middle-income countr*[TIAB]OR low income nation*[TIAB] OR middle income nation*[TIAB] OR low-income nation*[TIAB] OR middle-income nation*[TIAB] OR low income world[TIAB] OR middle income world[TIAB] OR low-income world[TIAB] OR middle-income world[TIAB] OR low income econom*[TIAB] OR middle income econom*[TIAB] OR low-income econom*[TIAB] OR middle-income econom*[TIAB] | 53,528 |
| 6 | LMIC[TIAB] OR LMICs[TIAB] OR LAMIC[TIAB] OR LAMICs[TIAB] OR LAMI countr*[TIAB] | 2,406 |
| 7 | Transitional countr*[TIAB] OR Transitional econom*[TIAB] OR Transition countr*[TIAB] OR Transition econom*[TIAB] | 331 |
| 8 | Asia[MH] OR Asia [TIAB] OR Africa[MH] OR Africa[TIAB] OR South America[MH] OR South America[TIAB] OR Caribbean region[MH] OR Caribbean[TIAB] OR Central America[MH] OR Central America[TIAB] | 1,129,233 |
| 9 | Afghanistan[TIAB] OR Algeria[TIAB] OR American Samoa[TIAB] OR Angola[TIAB] OR Antigua and Barbuda[TIAB] OR Aruba[TIAB] OR Argentina[TIAB] OR Armenia OR Azerbaijan[TIAB] OR Barbados[TIAB] OR Bangladesh[TIAB] OR Bahrain[TIAB] OR Belarus[TIAB] OR Byelarus[TIAB] OR Byelorussia[TIAB] OR Belorussia[TIAB] OR Belize[TIAB] OR Benin[TIAB] OR Bhutan[TIAB] OR Bolivia[TIAB] OR Botswana[TIAB] OR Brazil[TIAB] OR Brasil[TIAB] OR Burkina[TIAB] OR Burkina Faso[TIAB] OR Burundi[TIAB] OR Urundi[TIAB] OR Cambodia[TIAB] OR Republic of Kampuchea[TIAB] OR Cameroon[TIAB] OR Cameroons[TIAB] OR Cape Verde[TIAB] OR Central African Republic[TIAB] OR Chad[TIAB] OR Chile[TIAB] OR China[TIAB] OR Colombia[TIAB] OR Comoros[TIAB] OR Comoro Islands[TIAB] OR Comores[TIAB] OR Congo[TIAB] OR DRC[TIAB] OR Zaire[TIAB] OR Costa Rica[TIAB] OR Cote d'Ivoire[TIAB] OR Ivory Coast[TIAB] OR Cuba[TIAB] OR Djibouti[TIAB] OR Obock[TIAB] OR French Somaliland[TIAB] OR Dominica[TIAB] OR Dominican Republic[TIAB] OR Ecuador[TIAB] OR Egypt[TIAB] OR United Arab Republic[TIAB] OR El Salvador[TIAB] OR Equatorial guinea[TIAB] OR Eritrea[TIAB] OR Ethiopia[TIAB] OR Fiji[TIAB] OR French Guiana[TIAB] OR Gabon[TIAB] OR Gabonese Republic[TIAB] OR Gambia[TIAB] OR Georgia[TIAB] OR Ghana[TIAB] OR Gold Coast[TIAB] OR Grenada[TIAB] OR Guam[TIAB] OR Gibraltar[TIAB] OR Guadeloupe[TIAB] OR Guatemala[TIAB] OR Guinea[TIAB] OR Guinea-Bissau[TIAB] OR Guiana[TIAB] OR Guyana[TIAB] OR Haiti[TIAB] OR Honduras[TIAB] OR India[TIAB] OR Indonesia[TIAB] OR Iran[TIAB] OR Iraq[TIAB] OR Jamaica[TIAB] OR Jordan[TIAB] OR Kazakhstan[TIAB] OR Kenya[TIAB] OR Kiribati[TIAB] OR Kyrgyzstan[TIAB] OR Kirghizstan[TIAB] OR Kirgizstan[TIAB] OR Kirghizia[TIAB] OR Kirgizia[TIAB] OR Kyrgyz[TIAB] OR Kirghiz[TIAB] OR Kyrgyz Republic[TIAB] OR Republic of Korea[TIAB] OR Lao[TIAB] OR Laos[TIAB] OR Lebanon[TIAB] OR Lesotho[TIAB] OR Basutoland[TIAB] OR Liberia[TIAB] OR Libya[TIAB] OR Macao[TIAB] OR Madagascar[TIAB] OR Malagasy Republic[TIAB] OR Malawi[TIAB] OR Nyasaland[TIAB] OR Malaysia[TIAB] OR Malaya[TIAB] OR Malay[TIAB] OR Maldives[TIAB] OR Mali[TIAB] OR Marshall Islands[TIAB] OR Martinique[TIAB] OR Mauritania[TIAB] OR Mauritius[TIAB] OR Mayotte[TIAB] OR Mexico[TIAB] OR Micronesia[TIAB] OR Mongolia[TIAB] OR Morocco[TIAB] OR Mozambique[TIAB] OR Myanmar[TIAB] OR Burma[TIAB] OR Namibia[TIAB] OR Netherlands Antilles[TIAB] OR New Caledonia[TIAB] OR Nepal[TIAB] OR Nicaragua[TIAB] OR Niger[TIAB] OR Nigeria[TIAB] OR Oman[TIAB] OR Pakistan[TIAB] OR Palau[TIAB] OR Palestine[TIAB] OR Panama[TIAB] OR Papua New Guinea[TIAB] OR Paraguay[TIAB] OR Peru[TIAB] OR Philippines[TIAB] OR Puerto Rico[TIAB] OR Reunion[TIAB] OR Russia[TIAB] OR Russian Federation[TIAB] OR USSR[TIAB] OR Soviet Union[TIAB] OR Union of Soviet Socialist Republics[TIAB] OR Rwanda[TIAB] OR Ruanda-Urundi[TIAB] OR Samoa[TIAB] OR Samoan Islands[TIAB] OR Sao Tome[TIAB] OR Principe[TIAB] OR Senegal[TIAB] OR Seychelles[TIAB] OR Sierra Leone[TIAB] OR Solomon Islands[TIAB] OR Somalia[TIAB] OR South Africa[TIAB] OR Sri Lanka[TIAB] OR Ceylon[TIAB] OR Saint Kitts[TIAB] OR St Kitts[TIAB] OR Saint Christopher Island[TIAB] OR Nevis[TIAB] OR Saint Lucia[TIAB] OR St Lucia[TIAB] OR Saint Vincent[TIAB] OR Saudi Arabia[TIAB] OR St Vincent[TIAB] OR Grenadines[TIAB] OR Sudan[TIAB] OR Suriname[TIAB] OR Surinam[TIAB] OR Swaziland[TIAB] OR Syria[TIAB] OR Syrian Arab Republic[TIAB] OR Tajikistan[TIAB] OR Tadzhikistan[TIAB] OR Tadjikistan[TIAB] OR Tanzania[TIAB] OR Thailand[TIAB] OR Timor-Leste[TIAB] OR East Timor[TIAB] OR Togo[TIAB] OR Togolese Republic[TIAB] OR Tonga[TIAB] OR Trinidad and Tobago[TIAB] OR Tunisia[TIAB] OR Turkey[TIAB] OR Turkmenistan[TIAB] OR Turkmenia[TIAB] OR Tuvalu[TIAB] OR Uganda[TIAB] OR Uruguay[TIAB] OR Uzbekistan[TIAB] OR Vanuatu[TIAB] OR New Hebrides[TIAB] OR Venezuela[TIAB] OR Vietnam[TIAB] OR Viet Nam[TIAB] OR West Bank[TIAB] OR Gaza[TIAB] OR Yemen[TIAB] OR Zambia[TIAB] OR Zimbabwe[TIAB] OR Rhodesia[TIAB] | 898,971 |
| 10 | Europe[MH] OR Europe[TIAB] OR United states[MH] OR United states[TIAB] OR Canada[MH] OR Canada[TIAB] OR Australia[MH] OR Australia[TIAB] OR Europe*[TIAB] OR Australia*[TIAB] OR Albania[TIAB] OR Andorra[TIAB] OR Austria[TIAB] OR Belgi*[TIAB] OR Bosnia and Herzegovina[TIAB] OR Bulgaria[TIAB] OR Croatia[TIAB] OR Cyprus[TIAB] OR Czech Republic[TIAB] OR Denmark[TIAB] OR Finland[TIAB] OR France[TIAB] OR German*[TIAB] OR Greece[TIAB] OR Hungary[TIAB] OR Ireland[TIAB] OR Italy[TIAB] OR Kosovo[TIAB] OR Latvia[TIAB] OR Lithuania[TIAB] OR Luxembourg[TIAB] OR Macedonia[TIAB] OR Malta[TIAB] OR Moldavia[TIAB] OR Netherlands[TIAB] OR dutch[TIAB] OR Norway[TIAB] OR Poland[TIAB] OR Portugal[TIAB] OR Romania[TIAB] OR Slovak Republic[TIAB] OR Slovenia[TIAB] OR Spanish[TIAB] OR Spain[TIAB] OR Swed*[TIAB] OR Switserland[TIAB] OR Ukraine[TIAB] OR United Kingdom[TIAB] OR Brit*[TIAB] OR Yugoslavia[TIAB] | 3,230,772 |
| 11 | 1 OR 2 OR 3 OR 4 OR 5 OR 6 OR 7 OR 8 OR 9 NOT 10 | 1,353,879 |
| *Health insurance* | | |
| 12 | Insurance, health[MH] OR Insurance, health[TIAB] OR Health Insurance[TIAB] OR Health Insurance, Voluntary[TIAB] OR Insurance, Voluntary Health[TIAB] OR Voluntary Health Insurance[TIAB] OR Group Health Insurance[TIAB] OR Health Insurance, Group[TIAB] OR Insurance, Group Health[TIAB] | 158,693 |
| 13 | Health financing[TIAB] OR Health care insurance[TIAB] OR Medical insurance[TIAB] OR Medical coverage[TIAB] OR Social insurance[TIAB] OR Health coverage[TIAB] OR (subsidized[TIAB] AND healthcare[TIAB]) OR (health[TIAB] AND discounts[TIAB]) | 8,539 |
| 14 | Private health insurance[TIAB] OR social health insurance[TIAB] OR community based health insurance[TIAB] OR CBHI[TIAB] OR microinsurance[TIAB] OR micro-insurance[TIAB] OR micro insurance [TIAB] OR prepaid health plans[MH] OR prepaid health plans[TIAB] OR Insurance Coverage[TIAB] | 23,671 |
| 15 | 12 OR 13 OR 14 | 165,839 |
| *Vulnerable groups* | | |
| 16 | Minority groups[MH] OR Minority groups[TIAB] OR vulnerable populations[MH] OR vulnerable populations[TIAB] OR (Social class[MH] and Minority groups[MH]) OR underserved population[TIAB] OR disadvantaged[TIAB] OR population at risk[TIAB] OR marginali*[TIAB] OR Social inclusion[TIAB] | 45,083 |
| 17 | Female-headed household*[TIAB] OR female headed household[TIAB] OR widow*[TIAB] OR ((single headed households) AND (women[TIAB] OR gender[TIAB])) | 8,114 |
| 18 | Disabled children[MH] OR Disabled children[TIAB] OR child orphaned[MH] OR child orphaned[TIAB] OR children with special needs[TIAB] OR Cerebral palsy[MH] OR Cerebral pals*[TIAB] OR intellectual impair*[TIAB] OR intellectual deficien*[TIAB] OR intellectual disable*[TIAB] OR intellectual disabili*[TIAB] OR intellectual handicap*[TIAB] OR Learning disorders[MH] OR Learning disorders[TIAB] OR learning disorder*[TIAB] OR communication disorders[MH] OR communication disorders[TIAB] OR communication disorder*[TIAB] OR Pervasive Child Development Disorders[MH] OR Pervasive Child Development Disorders[TIAB] OR autistic[TIAB] OR autism[TIAB] OR asperger*[TIAB] OR dyslexi*[TIAB] OR Down’s Syndrome[TIAB] OR Down Syndrome[TIAB] OR Mongolism[TIAB] or Trisomy 21[TIAB] | 161,438 |
| 19 | Aged[MH] OR aged p*[TIAB] elderly[TIAB] OR elder*[TIAB] OR frail elderly[TIAB] OR old age[TIAB] OR senior citizens[TIAB] OR seniors[TIAB] | 251,643 |
| 20 | Homeless youth[MH] OR Homeless youth[TIAB] OR youth[TIAB] OR youth with special needs[TIAB] OR young adult*[TIAB] OR Runaway youth[TIAB] OR Runaway adolescent[TIAB] | 132,161 |
| 21 | Minority groups[MH] OR Minority groups[TIAB] OR Ethnic minorities[TIAB] OR Racial minorities[TIAB] OR racial disparit*[TIAB] OR ethnic dispar*[TIAB] | 24,015 |
| 22 | Migrants[MH] OR Migrants[TIAB] OR Transients and migrants[MH] OR Transients and migrants[TIAB] OR migrant*[TIAB] OR displaced populations[TIAB] OR displaced population[TIAB] OR mobile population*[TIAB] OR unsettled population[TIAB] OR young migrant*[TIAB] | 20,129 |
| 23 | Chronic disease[MH] OR Chronic disease[TIAB] OR chronic illness[TIAB] OR chronically ill[TIAB] OR suffering from chronic illness[TIAB] OR chronic disorder[TIAB] OR genetic diseases, inborn[MH] OR genetic diseases, inborn[TIAB] | 841,410 |
| 24 | Disabled person[MH] OR disabled person*[TIAB] OR disabled person*[TIAB] OR person with disabilit*[TIAB] OR persons with disabilit*[TIAB] OR people with disability*[TIAB] OR handicapped person*[TIAB] OR handicapped people[TIAB] OR Physical impair*[TIAB] OR physically impair*[TIAB] OR physical disab*[TIAB] OR physically disab*[TIAB] OR physical handicap*[TIAB] OR physically handicap*[TIAB] OR physically challeng*[TIAB] OR mentally disabled person[MH] OR mentally disabled person[TIAB] OR mentally impair*[TIAB] OR Cerebral palsy[MH] OR cerebral palsy[TIAB] OR spinal dysraphism[MH] OR spinal dysraphism[TIAB] OR spina bifida[TIAB] OR muscular dystrophies[MH] OR muscular dystroph*[TIAB] OR Arthritis[MH] OR Arthriti*[TIAB] OR Musculoskeletal Abnormalities[MH] OR Musculoskeletal Abnormalities[TIAB] OR skeletal abnormal*[TIAB] OR limb abnormalit*[TIAB] OR amputation[TIAB] OR Chronic brain injury[MH] OR chronic brain injury[TIAB] OR clubfoot[TIAB] OR Poliomyelitis[MH] OR poliomyelitis[TIAB] OR polio*[TIAB] OR Paraplegia[MH] OR Parapleg*[TIAB] OR paralyz*[TIAB] OR Hemiplegia[MH] OR Hemipleg*[TIAB] OR Hearing loss[MH] OR Deaf[TIAB] OR hearing disabili*[TIAB] OR Blindness[MH] OR Blindness[TIAB] OR visually impair*[TIAB] OR blind[TIAB] NOT double blind*[TIAB] OR Mental disorder*[TIAB] OR schizophrenia and disorders with psychotic features[MH] OR schizophrenia and disorders with psychotic features[TIAB] OR Dementia*[TIAB] OR Alzheimer*[TIAB] OR intellectual illness*[TIAB] OR intellectual impair*[TIAB] OR intellectual disab*[TIAB] OR intellectual handicap*[TIAB] OR mental ill[TIAB] OR mentally ill[TIAB] OR mental illness*[TIAB] OR mental impair*[TIAB] OR mentally impair*[TIAB] OR mental disab*[TIAB] OR mentally disab*[TIAB] OR mental handicap*[TIAB] OR mentally handicap*[TIAB] OR developmental impair*[TIAB] OR developmentally disabili*[TIAB] OR psychological ill*[TIAB] OR psychological disable*[TIAB] OR psychologically disable*[TIAB] OR psychological disabili*[TIAB] OR psychological handicap*[TIAB] OR psychologically handicap*[TIAB] OR Learning disorders[MH] OR learning disorder*[TIAB] OR communication disorders[MH] OR communication disorder*[TIAB] | 1,148,202 |
| 25 | 16 OR 17 OR 18 OR 19 OR 20 OR 21 OR 22 OR 23 OR 24 | 2,319,386 |
| 26 | 10 AND 15 AND 25 | 2402 |
| 27 | Limit 26 to English language, 1995-current, publication type= classical article, clinical trial, comparative study, journal article, evaluation studies, meta-analysis, observational study, randomized controlled trial | 2095 |
