# Supplementary material for: Leaving no one behind? Social inclusion of health insurance in low- and middle-income countries: a systematic review
Source: Int J Equity Health. 2019 Aug 28;18:134. doi: 10.1186/s12939-019-1040-0 (PMC6714392; doi:10.1186/s12939-019-1040-0)
Supplement: Supplementary file 2 — Included studies. Summary findings from included studies. (DOCX 147 kb) [file 12939_2019_1040_MOESM2_ESM.docx]

Additional file 2. Included studies*

*The table is organized per vulnerable group, per continent, per country (alphabetical), type of health insurance, within one country per scheme (alphabetical), and within one scheme per name of first author (alphabetical).

** RCT is randomized controlled trial design; QEx is Quasi-experimental design; Obs is observational design; Qual is qualitative design.

*** Only includes studies of medium quality of higher. See text for more detail on quality assessment.

**** In the table, + denotes a positive effect, 0 denotes no effect and - denotes a negative effect. ^ is not statistically significant in the particular study.

Abbreviations:

SHI: Social health insurance scheme

PHI: Private health insurance scheme

CBHI: Community based health insurance scheme

OOP: Out of pocket payments

HI: health insurance

CHE: catastrophic health expenditures

SES: socio-economic status

CASP: critical appraisal skills programme

| Scheme (SHI, PHI, CBHI, mixed) | Country | Reference | Vulnerable group included | Study type ** | Study context, recruitment and sample | Quality grade  (CASP)*** | Comparison group | Enrolment **** | Utilization **** | Financial protection **** | Health outcomes **** | Quality of care **** | Main results | Enablers / Barriers (for enrolment (E), utilization (U), impact (I) |
| --- | --- | --- | --- | --- | --- | --- | --- | --- | --- | --- | --- | --- | --- | --- |
| **Chronically ill** | | | | | | | | | | | | | | |
| *Africa* | | | | | | | | | | | | | | |
| CBHI | Ethiopia | (Mebratie et al. 2015) | Chronically ill, measure: family >6 years members suffering from symptoms > 30 days | Obs Longitudinal household survey | 1,224 households in 2011, 1,203 in 2012, 87% is male headed household. | Medium 8/11 points,  73% | General population | 0 |  |  |  |  | Chronically ill are less insured (in descriptive data) compared to population groups that are not chronically ill, though probability of enrolment for these groups shows no significant effect. | Enabling (E): premium low compared to user fee, pressure from CBHI officials  Barriers: registration fee not affordable lack of awareness about scheme. |
| NHIS (Ghana) (SHI) | Ghana | (Parmar et al. 2014b) | Chronically ill, who are 60 years and above, chronic illness diagnosed by health care provider | Obs, cross sectional household survey | Sampled by consulting Ghana Statistical Service to ensure a mix of urban and rural areas, in each district, nationally representative enumeration areas were selected, n=435, 51.9% female | Medium 10/11 points, 91% | Having no chronic disease | + |  |  |  |  | Those who are vulnerable in political dimension are less likely to enroll in NHIS. Older adults with chronic disease are significantly more likely to enroll in NHIS. | Enabling (E): being hospitalized, belonging to majority religious group, barrier: living far from health facilities. |
| Mixed schemes: SHI, PHI, CBHI, Pre-payment scheme or other | Kenya | (Kazungu & Barasa 2017) | Chronically ill, measure hypertension or diabetes | Obs, cross sectional | Secondary data from Kenya Demographic Health survey in 2009 and 2014, highest age in sample is 49 years. | Medium 11/14, points,  79% | Absence of having a chronic disease | + |  |  |  |  | Chronically ill had, despite having a borderline significance 22% greater odds of coverage compared to those without a chronic disease. |  |
| CBHI (Mutual de Santé) | Rwanda | (Finnoff 2016) | Chronically ill (women, not indicated how illness is assessed) | Obs, cross sectional | EICV2 (see reference in article), employed a two-stage stratified sample design, with a national sample of 34,785 individuals in 6,900 households. | Medium 9/11 points,  82% | Other subgroups | 0 |  |  |  |  | Gender had no significance in determining use of health care service. Those individuals reporting a chronic illness or disability do not have any difference in MHI enrollment, compared to other subgroups. |  |
| Plan Sesame (SHI) | Senegal | (Parmar et al. 2014b) | Chronically ill, who are 60 years and above, chronic illness diagnosed by health care provider | Obs, cross sectional household survey | 4 regions, list of enumeration areas was used, to select proportionally the number of households heterogeneous setting (rural urban, several regions), n=2917, 46.6% female | Medium 10/11 points, 91% | Having no chronic disease | + |  |  |  |  | Vulnerable older adults in all dimensions (social, political, economic and cultural) are less likely to enroll in Plan Sesame. Older adults with chronic disease significantly more likely to enroll in Plan Sesame. | Enabling (E): being hospitalized, men, household head, having formal education, urban area, majority ethnicity. Barriers (E): lower political participation, unsafe environment, limited access to information |
| Community health fund (CBHI) | Tanzania | (Macha et al. 2014) | Chronically ill, not indicated how assessed | Qualitative | 12 focus group discussions (FGD), with insured non-insured and dropouts | Medium 8/10 points,  80% | Other groups, not specifically indicated | + ^ |  |  |  |  | Members with chronic diseases were joining the scheme more than other groups (push factor), not significant, (qualitative study). | Enabling (E): higher income, having an occupation, large household size, poor health status, high level of understanding of risk pooling |
| *Asia* | | | | | | | | | | | | | | |
| New cooperative medical scheme (NCMS) (SHI) | China | (Che et al. 2016) | Chronically ill, hepatitis B virus-related disease | Obs, cross sectional survey | Sample from one region, 940 hepatitis b infected patients, mean age 44, male to female ratio 2:1. | Medium 10/11 points, 91% | Several groups having chronic disease due to hepatitis B | - |  | - |  |  | Patients with early stage liver diseases are least assisted with health insurance as they are mostly outpatients, compared to other groups having chronic diseases due to hepatitis B. UEM and URM (see reference below) prevent more patients from catastrophic health expenditures than NCMS. Not clear what effect is compared to non-insured. |  |
| NCMS (SHI) | China | (Jiang et al. 2012) | Chronically ill, as in diabetes, hypertension, heart disease, malignant tumor or chronic obstructive pulmonary disease | Obs, cross sectional survey | National health services survey, 2008, rural sample 13,990 subjects. | Medium 10/11 points, 91% | Individuals without a chronic disease |  |  | - |  |  | Those with a chronic patient in the family had higher risk of catastrophic health expenditures than those without. CATAplusA and CATAplusB offered weaker financial protection than Cata. | In general (total population): Cata provided significant financial protection compared to non-insured. |
| NCMS (SHI) | China | (Jing et al. 2013) | Chronically ill, self-identified illness that continues at least 3 months or intermitted presentation within 12 months | Obs, cross sectional survey | Household survey, multistage sampling per county (3): 3 townships, 6 villages, 60 households per village. N= 2998 followed in 2 years, household heads: 92.4% were men. | Medium  8/11 points, 73% | Counties where the newly implemented re-imbursement policies did not yet take place |  |  | + ^ |  |  | At all, having the NCMS scheme does decrease catastrophic health expenditures for chronically ill, however after controlling for household demographics not significant, compared to the control group without newly implemented reimbursement policies. Those policies for chronic disease in the NCMS programs were not significantly effective in reducing household CHI. | Enabling factor (I) on financial protection: household gross income per capita |
| NCMS (SHI) | China | (Sun et al. 2009) | Chronically ill, self-identified illness that continues at least 3 months or intermitted presentation within 12 months | Obs, sub data base analyses | Chronic disease patients, six counties, 6,147 households, including 3,944 individuals with chronic diseases. | Medium 8/11 points, 73% | Non-members | + | - | + ^ |  |  | Chronic disease prevalence was higher among NCMS members than non-members. Proportion of patients covered by NCMS who did not seek treatment was higher than that of non-members. Lower catastrophic expenditures than non-members but not sign., reimbursement rate very low (9-11% of overall medical expense) | Barrier (E): Being poor, being male |
| NCMS (SHI) | China | (Wang et al. 2014) | Chronically ill, individuals reported to have been diagnosed with diabetes, hypertension, heart disease, malignant tumor, rheumatic arthritis | Obs, comparative, cross sectional | Multi stage stratified random sampling, 1,800 questionnaires sent, 1,661 returned, 1,525 analyzed. | Medium 8/11 points, 73% | Households without an individual suffering from chronic disease |  |  | + ^ |  |  | After health care expenditure poverty prevalence increase, then after NCMS reimbursement, prevalence of poverty and CHE decrease only slightly. After reimbursement prevalence of poverty and CHI among chronically ill was higher than those without chronic disease. | Enabling for financial protection: higher income |
| SHI (not indicated which schemes) | China | (Liao et al. 2016) | Chronically ill, individuals above 35 with hypertension | QEx, before after design, repeated cross sectional design | Subjects with hypertension, controlled with subjects with normal bp. N=9971, 51.6% female. | High 10/12 points, 83% | Subjects with normal blood pressure (BP). |  | + |  |  | + | Health insurance showed a positive effect on treatment, subjects with health insurance had a 29% higher chance to receive hypertension treatment. QoC: Awareness on high BP increased. |  |
| Urban Employees Medical scheme (UE-BMI) (SHI)  Urban Residents Medical scheme (UR-BMI) (SHI) | China | (Che et al. 2016) | Chronically ill, hepatitis B virus-related disease | Obs, cross sectional survey | Sample from one region, 940 hepatitis b infected patients (HBV), having acquired chronic diseases, mean age 44, male to female ratio 2:1. | Medium 10/11 points, 91% | Several groups having chronic disease due to hepatitis B. |  |  |  |  |  | Patients with early stage liver diseases are least assisted with health insurance as they are mostly outpatients, compared to other chronic diseases due to hepatitis B. UEM and URM prevent from catastrophic health expenditures than NCMS. |  |
| Public HI; Private HI; Public + private; Public rural and public urban, no insurance (mixed) | China | (Jin et al. 2016) | Chronically ill, having been diagnosed by one of the listed diseases | Obs, longitudinal survey | Representative sample of individuals who are aged 45 and above and their spouses, n=17,711 in 2011 and n=18,618 in 2013. | Medium 8/11 points, 73% | Several insurance types and no insurance | - |  |  |  |  | Chronically ill were less likely to purchase private insurance and more likely to have no insurance. Only urban residents with chronic illness were more likely to have private insurance. |  |
| SHI and PHI (mixed) | China | (Lam & Johnston 2012) | Chronically ill | Obs, cross-sectional survey | 4 groups, noninsured, SHI, SHI+ PHI and PHI. | Medium 8/11 points,  73% | Registered citizens | 0 |  |  |  |  | No effect for chronically ill rural-urban migrants for uptake of HI. |  |
| Rashtriya Swasthya Bima Yojna (RSBY) (SHI) | India | (Philip et al. 2016) | Chronically ill | Obs, comparative cross-sectional survey in one district | 3 stage random sampling, 149 insured and 147 non-insured, no matching | Medium 8/11 points, 73% | Having no chronic disease | + | + |  |  |  | Insured households had higher number of individuals with chronic diseases (adverse selection). Pre-existing chronic disease was a reason to use inpatient services more frequent |  |
| Jamkesmas (SHI) | Indonesia | (Aji et al. 2014) | Chronically ill, households that have faced illness that resulted in high expenses | Qualitative, in-depth interviews with open ended and semi-structured questions | Qualitative study, using purposive sampling, in one district, data from one hospital, 73% female. | Medium  8/10 points, 80% | Households whose families were not hospitalized |  |  | 0 |  |  | Jamkesmas insurance holders are not required to cost share or pay for other medical expenses during hospital stays. |  |
| Jamsostek Askes (SHI) | Indonesia | (Aji et al. 2014) | Chronically ill, households that have faced illness that resulted in high expenses | Qualitative, in-depth interviews with open ended and semi-structured questions | Qualitative study, using purposive sampling, in one district, data from one hospital, 73% female. | Medium 8/10 points, 80% | Households whose families were not hospitalized |  |  | - |  |  | Askes and Jamsostek schemes experience higher direct and indirect costs than Jamkesmas (the above). Having a family member hospitalized for chronic illness affected households’ financial resources. | Barrier for financial protection: Inappropriate benefit packages. |
| CBHI (grass root organizations for financial protection) | Philippines | (Dror et al. 2005) | Chronically ill, in total and subgroup diabetes and hyper-tension | QEx | Two stage cluster sampling method, pooled data. 1953 households, 890 insured, 1063 non-insured. 0.99 males to 1 female, 22 years is median age in both groups. | High  8/12 points, 66% | Non-insured | + ^ | + |  |  |  | More chronic illness was diagnosed in the insured group (not significant). Insured chronically ill have higher drug compliance. Results not generalizable due to various schemes in one group of ‘insured’ in this study. |  |
| Micro health finance (CBHI) | Syria | (Daher & Flessa 2010) | Chronically ill, patients with lung disease | Obs, cross sectional | Patients recruited from hospital admissions, 90 persons with lung disease (average age 43.8). | Medium 8/11 points, 73% | Individuals with disabilities, having a walking disability | - |  | - |  |  | 63% of patients with lung diseases, who were not able to work had no access to the financial service (enrollment) and were rejected from micro financing.  Treatment cost in private hospital, drugs, medical devices were not or not fully covered by HI. Loans for medical devices also not included. | 95% of the individuals believed microfinance would be an ideal tool to get devices that enable them to work, and then, being able to pay back. |
| Social health insurance (SHI) | Vietnam | (Nguyen et al. 2017) | Chronically ill, HIV/AIDS patients | Obs, cross sectional | 3 hospitals and 5 outpatient clinics in 2 provinces, convenience sample n=1,133, average age 35.5, male: 58.7% | Medium 8/11 points, 73% | Other people having no health insurance | -^ |  | - |  |  | Low rate of insurance among people living with HIV/AIDS, rural enrolment higher than urban, no comparison with GP. Inpatient service usage in city was 2x higher than rural. Likelihood of having HI when facing CHE was 1.79 times higher than other people | Enabling (E): higher educated, living with spouse. Barriers (E): lack of info about HI, difficult access to HI and to pay HI, living rural. |
| *South America* | | | | | | | | | | | | | | |
| FONASA (SHI) | Chile | (Bitran et al. 2010) | Chronically ill, hypertension, diabetes type 1 or type 2, depression, epilepsy, HIV/AIDS | Obs, cohort, longitudinal study | Secondary data of national health survey on chronic disease, n differs per chronic disease, from 1,2 million to 2,978 patients | Medium 8/11 points, 73% | Among these types of diseases, comparison in time |  | + |  |  |  | Increase use of chronic care services and hospital care and reduced mortality once enrolled for persons having one of the six most common chronic diseases. Hospitalization rates drop due to increased access to health. | Barrier on QoC: access to care was restricted by waiting lists than in ISAPRE, more complaints about waiting list |
| FONASA (SHI) | Chile | (Castillo-Laborde et al. 2017) | Chronically ill | Obs | 13,451,188 enrollees for FONASA. Data from several resources, including health insurance agencies, comparative study with private scheme. | Medium 9/11 points, 82% | Private insurance | 0 | - | - |  |  | More admissions that could have been prevented by outpatient services. FONASA has higher incidence of catastrophic expenditures due to OOP and less beds for FONASA insured with chronic diseases |  |
| ISAPRE (SHI) | Chile | (Bitran et al. 2010) | Chronically ill, hypertension, diabetes type 1 or type 2, depression, epilepsy, HIV/AIDS | Obs | Secondary data of national health survey on chronic disease, n differs per chronic disease, from 1,2 million to 2,978 patients | Medium  8/11 points, 73% | Among these types of diseases, comparison in time |  | + |  | + |  | Increase use of chronic case services and hospital care and reduced mortality once enrolled for persons having one of the six most common chronic diseases. |  |
| ISAPRE (SHI) | Chile | (Castillo-Laborde et al. 2017) | Chronically ill, 18 specific chronic conditions | Obs | 3,206,312 Individuals included in study. Data from several resources, including health insurance agencies. | Medium  9/11 points, 82% | Private insurance | 0 | + |  |  | + | Age adjusted prevalence of chronic disease was not significantly higher in ISAPRE beneficiaries.  ISAPRE members with chronic illness make more use of specialized care. QoC: ISAPRE members have higher user satisfaction. |  |
| Seguro Popular health insurance (SPHI) (SHI) | Mexico | (Doubova et al. 2015) | Chronically ill, diagnosis of a chronic disease | QEx, case control study, score matching analysis with non-insured | Based on secondary data from the 2012 Mexican Survey of Health and Nutrition. 18,847 older adults from 13,690 households. | High, 10/12 points, 83% | Non-insured and pre-existing social security health insurance | + | + | - |  |  | Compares SPHI and SSHI with those without HI. SPHI showed a protective effect against lack of access to health care for Mexican older adults with chronic illness.  SPHI individuals use more savings, borrowing money or selling assets to access care | Enrolment: SPHI insured more often have a lower SES, and barriers: distances to health services and lack of satisfaction of providers’ behavior. |
| SPHI (SHI) | Mexico | (Rivera-Hernandez et al. 2016) | Chronically ill, diabetes and hypertension | Obs, cross sectional | National health surveys, 3 times of data collection (2000, ‘06, ‘12), n=3015 with diabetes, n=5307 with hypertension | Medium, 8/11 points, 73%. | Among these types of diseases, comparison in time | + | + for diabetes  0 for hyper-tension |  |  |  | Enrollment rates among diabetic and hypertension raised significantly. For those with diabetes, increased medication utilization. |  |
| Seguro popular (SP) and employer-based insurance (IMSS, ISSSTE, PEMEX, private HI | Mexico | (Rivera-Hernandez & Galarraga 2015) | Chronically ill, screening for hypertension, cholesterol, diabetes, prostate cancer, cervical cancer | Obs, cross sectional | National health survey, n=46,277, of whom 11,464 were 50 years old and above with complete demographic data, who were slightly older 56% were female, 12% spoke indigenous language | Medium, 9/11 points,  82% | Non- insured | + | + |  |  |  | More members had chronic disease among employment-based insurance and SP than non-insured. SP enrollees and employer-based enrollees were more likely to use screening tests, except for cervical cancer. |  |
| Any type of HI (mixed schemes) | Mexico | (Maurer 2008) | Chronically ill, arthritis, diabetes, heart attack, hypertension, respiratory disease or stroke. | Obs, cross sectional | MHAS is nationally representative sample of older Mexicans (50+), n=14,397, 57% is female, 55% has any health condition (HC), 21% has multiple HCs. | Medium 9/11 points,  82% | Among several types of disease and several types of insurance |  | + |  |  |  | Having any HI increased probability of medication treatment for chronically ill. Mexicano del Seguro Social (IMSS) had the largest effect, designed for formal workers of private companies. |  |
| *Multi-continent* | | | | | | | | | | | | | | |
| Any type of Health insurance (mixed schemes) | China Ghana  India  Mexico Russian federation South-Africa | (Goeppel et al. 2016) | Older adults (50+) with at least one chronic disease | Obs, cross sectional | Sage study, household survey of global ageing and adult health, n=16.631, response rate 52 to 93 %. | Medium 9/11 points, 82% | Non-insured |  |  | - |  |  | Insured households had higher catastrophic expenditures during last year, perhaps due to more frequent service utilization than the non-insured. Inequity of access was not fully determined by the level of economic development or insurance coverage. |  |
| Any scheme (PHI, SHI, or CBHI) | 48 LMIC | (El-Sayed et al. 2015) | Chronically ill, high-burden NCDs (any diagnosis, angina, asthma, depression, arthritis, schizophrenia, or diabetes | QEx | Several household surveys in many LMIC, data from WHO 2002-2004, n=197,914. | High 10/12 points,  84% | Non-insured | + | + | + |  |  | Insurance may be an important tool to increase Non-communicable disease treatment and protect against the harmful financial consequences of illness. Enrolment rates are higher for chronically ill. With uninsured status individuals are less likely to receive treatment, except for diabetes. Insurance predicted lower likelihood of borrowing or selling to pay for health services. | Enhancing factors (E): being unmarried, having secondary education and greater household wealth. Poor women have lowest likelihood of receiving treatment. |
| National (social) insurance (SHI) or private (PHI) | Ghana  Uganda, Kenya Philippines Jordan | (Vialle-Valentin et al. 2015) | Chronically ill, hypertension, diabetes, hypercholesterolemia, chronic heart disease, asthma, arthritis, chronic gastric pain, more than one chronic disease | Obs | WHO household survey, individuals with chronic disease: Ghana: n=165, 65% female (f), Kenya: n=313 54% f., Uganda: n=321 58% f., Jordan: n=583, 47% f., Philippines: n=485 52% f. | Medium 8/11 points, 73% | No (partial) insurance coverage |  |  |  |  | + | Enrolment in national/social HI varies from 75% in Jordan, 66% in Ghana, to 38% in Jordan and 22% in Kenya (Uganda not assessed). Likelihood of having access to medicine for chronic diseases was higher for those with medicine insurance coverage |  |
| Scheme (SHI, PHI, CBHI, mixed) | Country | Reference | Vulnerable group included | Study type ** | Study context, recruitment and sample | Quality grade  (CASP)*** | Comparison group | Enrolment **** | Utilization **** | Financial protection **** | Health outcomes **** | Quality of care **** | Main results | Enablers / Barriers (for enrolment (E), utilization (U), impact (I) |
| **Older adults** | | | | | | | | | | | | | | |
| *Africa* | | | | | | | | | | | | | | |
| Assurance Maladie a Base Communautair (AMBC) (CBHI) | Burkina Faso | (Parmar et al. 2014a) | Older adults, aged 61 and above | Obs, cross-sectional | Household survey, 2 stage cluster sampling, data from 2004 to 2008, 990 households. | Medium 10/14 points  71% | Individuals who are 60 and below | 0 | 0 |  |  |  | Older adults had no significant higher or lower odds to enrollment and utilization of health care. |  |
| CBHI | Ethiopia | (Mebratie et al. 2015) | Older adults, aged above 64 | Obs  Longitudinal household survey | 1,224 households in 2011, 1,203 in 2012, 87% is male headed household. | 8/11 points  73%  Medium | Younger population groups | 0 |  |  |  |  | Older adults are less insured (in descriptive data), though probability of enrolment for these groups shows no significant effect | Enabling (E): premium low compared to user fee, pressure from CBHI officials  Barriers: registration fee not affordable lack of awareness about scheme. |
| NHIS (SHI) | Ghana | (Duku et al. 2015) | Older adults, in 2 subgroups: 60-69 and 70+. | Obs, cross sectional | Households from 64 health facilities within 10km radius.  4,214 individuals above 18, including groups of 60-69, 70+ | Medium 8/11 points,  73% | Younger population groups | + | + | + for 70+  - for 60-69 |  | - | Enrolment: Higher enrolment for the 70+ and 60-69 groups. Utilization: 100% of 70+ used the primary health care services more than 10 times for the last 6 months. Financial protection: 60-69 age group could not afford the premium. Exemption fees have positive effect on enrolment for 70+.  QoC: low confidence in scheme. | Enabling factor (E): belonging to community group, being a man, among older adults. Barrier (E): low level of awareness about exemption policy in 70+ age group. |
| NHIS (SHI) | Ghana | (Fenny 2017) | Older adults, aged 60-69 and 70+ | Obs, cross sectional | Survey in 3 districts, multistage systematic sampling, randomly selected and stratified to urban and rural. 49% were aged 60-69 and 51% were 70+. | Medium 8/11 points, 73% | Older adults who are not insured | + for 70+  - for 60-69. |  |  |  |  | Individuals aged 60-69 are the least likely to have health insurance coverage and seem most financially vulnerable for enrollment, compared to exempted 70+. | Enabling (E): for 70+: fee exemption  60-69: being educated, living near health facility, being employed |
| NHIS (SHI) | Ghana | (Parmar et al. 2014b) | Older adults, who are vulnerable and aged over 70 | Obs, cross sectional household survey | Sampled by consulting Ghana Statistical Service to ensure a mix of urban and rural areas, in each district, nationally representative enumeration areas were selected, n=435, 51.9% female | Medium 10/11 points,  91% | Those who are not vulnerable according to SPEC framework | - |  |  |  |  | Those who are vulnerable in political dimension are less likely to enroll in NHIS. | Enabling (E): being hospitalized, belonging to majority religious group, barrier: living far from health facilities. |
| Plan Sesame (Senegal) (SHI) | Senegal | (Parmar et al. 2014b) | Older adults, vulnerable older adults who are 60 years and above | Obs, cross sectional household surveys, | Sampled by consulting Ghana Statistical Service to ensure a mix of urban and rural areas, in each district, nationally representative enumeration areas were selected, n=435, 51.9% female | Medium 10/11 points, 91% | Those who are not vulnerable according to SPEC framework | - |  |  |  |  | Vulnerable older adults in all dimensions (social, political, economic and cultural) are less likely to enroll in Plan Sesame. | Enabling (E): being hospitalized, men, household head, having formal education, urban area, majority ethnicity. Barriers (E): lower political participation, unsafe environment, limited access to information |
| Community health fund (CBHI) | Tanzania | (Macha et al. 2014) | Older adults, age limit not indicated | Qualitative | 12 focus group discussions (FGD), with insured non-insured and dropouts | 8/10 points  80%  Medium | Other groups, several subgroups based on socio demographics | + ^ |  |  |  |  | Older members were joining the scheme more than other groups (push factor), not significant (qualitative study). | Enabling (E): higher income, having an occupation, large household size, poor health status, high level of understanding of risk pooling |
| *Asia* | | | | | | | | | | | | | | |
| NCMS (SHI) | China | (Cheng et al. 2015) | Older adults, aged 60 and above. | QEx case control study | Survey data in rural China, 22 provinces. Mean age 83.1, 42% male. Treated are the individuals who enrolled in NCMS between 2005 and 2008 and controls are not insured. | High 10/12 points 83%. | Non-insured |  | + | 0 | + |  | Access to health care improves due to NCMS. Once enrolled there is improved ADL and cognitive functions of older adults and reduced likelihood of reduced blood level, especially for the poorest. No effect of NCMS reducing enrollees’ total medical expenditure and OOP. | Enabling: being married, lower income and more adult children alive for enrollees. Barrier: having less cognitive function. |
| SHI (UE-BMI UR-BMI and NCMS) | China | (Gu et al. 2017) | Older adults, aged 60 years and above | Obs, national baseline survey cross sectional comparing groups | Multistage sampling, 4,156 subjects (aged 60 years and above). | Medium 9/11 points, 82% | Various types of health insurance, or without insurance |  |  |  | + |  | All 3 types of health insurance have significant positive influence on health status of older adults. Health status of UR-BMI insured improved mostly, followed by UE-BMI and NCMS. | Enabling factors on health status: having proper leisure activities. Barrier is being an older woman, widow, having low income. |
| Public HI; Private HI; Public + private; Public rural and public urban, no insurance (mixed) | China | (Jin et al. 2016), | Older adults, aged 45 and above (minimum retirement age) | Obs, longitudinal survey | Representative sample of individuals who are aged 45 and above and their spouses, n=17,711 in 2011 and n=18,618 in 2013. | Medium 8/11 points 73% | Several insurance types and no insurance | - |  |  |  |  | Older people were less likely to purchase private insurance and more likely to have no insurance. |  |
| PHI, SHI, CBHI (mixed) | India | (Chakrabarti & Shankar 2015) | Older adults, aged 60 and above | Obs, empirical analysis in cross sectional study | Data from national family health survey 2005-2006, with 52,669 observations in rural areas and 40,591 observations in urban sample in 29 states in India. | Medium 10/11 points  91% | Households not having a higher number of older members | 0 |  |  |  |  | Households having a higher number of older members did not have a higher enrolment pattern. For all health insurance schemes. CBHI is not effective in attracting households with a more dependent population. | Enabling (E): Living in urban area: odds for enrolling in PHI or public HI in comparison to no HI are higher. |
| Various social health insurances (Askes, Jamsostek,Askeskin Jamkesmas) (SHI) | Indonesia | (Christiani et al. 2017) | Older adults (women) | Obs, cross sectional | 1,400 adult women from four major cities, survey data | Medium 11/11 points, 100% | Younger individuals | + |  |  |  |  | Being older has positive effect on health insurance enrolment, rates of women aged 60 years or more were 12% higher than women aged 15-30. | Barrier (E): being poor  Enabling (E):  higher education, being older |
| *South America* | | | | | | | | | | | | | | |
| Private health insurance (PHI) | Brazil | (Blay et al. 2008) | Older adults, 60 years of age or older, mainly descendant of European migrants. | Obs, cross sectional | 4975 non-institutionalized older adults, multistage, random sampling from 9 homogeneous geographic zones in Rio Grande do Sul | Medium  10/11 points, 91% | Non-insured |  | + |  |  |  | Being insured increased access to care for older adults. Outpatient visits were 2,5 times more likely when insured. | Enablers: being female, older, (formerly) employed, and higher income to get HI and visits to outpatient department. |
| Seguro Popular health insurance (SPHI) (SHI) | Mexico | (Doubova et al. 2015) | Older adults, 60 years and above | QEx, case controle study, score matching analysis with non-insured | Based on secondary data from the 2012 Mexican Survey of Health and Nutrition. 18,847 older adults from 13,690 households. | High, 10/12 points, 83% | Non-insured and pre-existing social security health insurance | 0 | + | - |  |  | SPHI showed a protective effect against lack of access to health care for Mexican older adults.  SPHI individuals use more savings, borrowing money or selling assets to access care | SPHI insured more often have a lower SES, and barriers: distances to health services and lack of satisfaction of providers’ behavior. |
| Any type of HI (mixed schemes) | Mexico | (Maurer 2008) | Older adults (50+) | Obs, cross sectional | MHAS is nationally representative sample of older Mexicans (50+), n=14,397, 57% is female, 55% has any health condition (HC), 21% has multiple HCs. | Medium 9/11 points,  82% | Among several types of disease and several types of insurance |  | + |  |  |  | Having any HI increased probability of medication treatment for chronically ill above 50 years. |  |
| Seguro popular (SP) and employer-based insurance (IMSS, ISSSTE, PEMEX, private HI (mixed) | Mexico | (Rivera-Hernandez & Galarraga 2015) | Older adults (50+) | Obs, cross sectional | National health survey, n=46,277, of whom 11,464 were 50 years old and above with complete demographic data, who were slightly older 56% were female, 12% spoke indigenous language | Medium, 9/11 points,  82% | Non-insured | + | + |  |  |  | More members had chronic disease among employment-based insurance and SP than non-insured. Use of preventive service among older adults being insured was significantly higher than those who are not insured. |  |
| *Multi-continent* | | | | | | | | | | | | | | |
| Any type of Health insurance | China Ghana  India  Mexico Russian federation South-Africa | (Goeppel et al. 2016) | Older adults (50+) with at least one chronic disease | Obs, cross sectional | Sage study, household survey of global ageing and adult health, n=16.631, response rate 52 to 93 %. | Medium 9/11 points, 82% | Non-insured |  |  | - |  |  | Insured households had higher catastrophic expenditures during last year, perhaps due to more frequent service utilization than the non-insured. |  |
| Scheme (SHI, PHI, CBHI, mixed) | Country | Reference | Vulnerable group included | Study type ** | Study context, recruitment and sample | Quality grade  (CASP)*** | Comparison group | Enrolment **** | Utilization **** | Financial protection **** | Health outcomes **** | Quality of care **** | Main results | Enablers / Barriers (for enrolment (E), utilization (U), impact (I) |
| **Individuals with disabilities** | | | | | | | | | | | | | | |
| *Africa* | | | | | | | | | | | | | | |
| Private health insurance (PHI) | Kenya | (de Menil et al. 2014) | Individuals with disabilities, mental disorder, substance use or common mental disorder | Obs, cohort | 455 unique patients (mentally ill) seen in 12 months. Mean age 36,3. | Medium  11/14 points, 79%. | No comparison with general population or with public institutions |  | + |  |  | - | Positive association between private health insurance and quantity of care (utilization) for individuals with mental illness. Having no insurance means getting services for a lower price, uninsured pay but less. | QoC: having a severe mental disorder was associated with a slightly higher charge per day. |
| CBHI (Mutual de Santé) | Rwanda | (Finnoff 2016) | Women with disabilities measure of disability not indicated | Obs, cross sectional | EICV2 (see reference in article), employed a two-stage stratified sample design, with a national sample of 34,785 individuals in 6,900 households. | Medium 9/11 points,  82% | Other subgroups | 0 |  |  |  |  | Gender had no significance in determining use of health care service. Those individuals reporting a chronic illness or disability do not have any difference in MHI enrollment. |  |
| Community health fund (CBHI) | Tanzania | (Macha et al. 2014) | Individuals with disabilities, measure of disability not indicated | Qual | 12 focus group discussions (FGD), with insured non-insured and dropouts | 8/10 points  80%  Medium | Other groups, based on several socio demographic factors | + ^ |  |  |  |  | Individuals with disabilities were joining the scheme more than other groups (push factor), not significant, (qualitative study). | Enabling (E): higher income, having an occupation, large household size, poor health status, high level of understanding of risk pooling |
| *Asia* | | | | | | | | | | | | | | |
| SHI (not indicated) | China | (Huang et al. 2013) | Individuals with disabilities (lost all or part of ability to engage in certain activities as a result function loss | Obs, cross sectional | Population-based national survey from county to community level, sample of rural individuals with disabilities: 114,458, 50% male. | Medium 10/11 points, 91% | No comparison with non-disabled groups. | -^ |  |  |  |  | 28% of individuals with disabilities is insured. Not compared to other groups. Negative effect for pilot county with new policies (NPS) on enrolment. Interaction between policy and economic factors and the disability degree was not statistically significant. | Enabling factors to be enrolled: rehabilitation station is near, having higher household income, having less severe disability degree. |
| Micro health finance (CBHI) | Syria | (Daher & Flessa 2010) | Individuals with disabilities, having a walking disability | Obs, cross sectional | Patients recruited from hospital admissions, 90 persons with lung disease (average age 43.8). | Medium 8/11 points, 73% | Chronically ill, patients with lung disease | - |  | - |  |  | 80% of individuals with disabilities, who were not able to work had no access to the financial service (enrollment) and were rejected from micro financing.  Treatment cost in private hospital, drugs, medical devices were not or not fully covered by HI. Loans for medical devices also not included. | 89% of individuals believed microfinance would be an ideal tool to get devices that enable them to work, and then, being able to pay back. |
| Public health system. (CHI compulsory health insurance) voluntary health insurance VHI, health insurance for the poor HIP. (mixed) | Vietnam | (Palmer & Nguyen 2012) | Individuals with disabilities, in domain of mobility, hearing, speaking, learning, mental illness, learning difficulty, and vision | Qex | Data from Vietnam national health survey 2001-2002, n=4905, average 44 (vs 29 non-disabled), 56% is male. | High 10/12 points,  83% | Persons without disabilities | 0 | + | + |  |  | Individuals with disabilities had a similar rate of insurance membership compared to those without disabilities. Scheme membership differed by disability status. Schemes significantly increase probability of accessing public inpatient and outpatient care. Insured individuals with disabilities spent 84% less than those uninsured. | Enabling (U): being male, married, educated, having more household members, living in urban area (outpatient only), living in south central coast region (inpatient only).  Barrier (U): age, income, ethnic minority status (inpatient only) |
| Social health insurance (SHI) | Vietnam | (Palmer 2014) | Individuals with disabilities, in the domain of vision, hearing, concentrating, walking, self-care, and communication | Obs, cross-sectional | Household and community information Survey (VHLSS), n= 1,265 with co-variate matching between insured and uninsured. Disability measured as ‘a lot of difficulty’ or ‘can’t do’ in one of the six domains. | Medium 10/11 points, 91% | Other groups, based on socio demographics |  | + | - |  |  | Insured persons with disabilities increased in frequency and length of hospital visits, especially tertiary level hospitals. Compared to other groups persons with disabilities had the highest difference between insured and uninsured on length of hospital stay (increased 2-3 days). Inpatient expenditures per visit doubled and was higher than in other groups and higher as uninsured. Outpatient expenditures were lower due to lower medication and consultation expenditures, however not significant. In total CHE increased with 7% and poverty increased with 4% when taking account for health costs. |  |
| *South America* | | | | | | | | | | | | | | |
| Seguro Popular health insurance (SPHI) (SHI) | Mexico | (Doubova et al. 2015) | Older adults with disabilities, measured by physical or mental limitation, self-reported | QEx, case control study, score matching analysis with non-insured | Based on secondary data from the 2012 Mexican Survey of Health and Nutrition. 18,847 older adults from 13,690 households. | High, 10/12 points, 83% | Non-insured and pre-existing social security health insurance | + | + | - |  |  | SPHI showed a protective effect against lack of access to health care for Mexican older adults with disabilities.  SPHI individuals use more savings, borrowing money or selling assets to access care | SPHI insured more often have a lower SES, and barriers: distances to health services and lack of satisfaction of providers’ behavior. |
| Social security and other (SHI) | Peru | (Bernabe-Ortiz et al. 2016) | Individuals with disabilities, permanent limitation on movement, vision, communication, hearing, learning/remembering or social relationships | Obs, cross sectional | Nationwide survey, rural and urban settings in and around several Peruvian cities | Medium 9/11 points, 82% | Non-insured or CBHI |  | + |  |  |  | Individuals with disabilities being enrolled in SHI have better access health care and rehabilitation services than those with no insurance or CBHI. |  |
| Scheme (SHI, PHI, CBHI, mixed) | Country | Reference | Vulnerable group included | Study type ** | Study context, recruitment and sample | Quality grade  (CASP)*** | Comparison group | Enrolment **** | Utilization **** | Financial protection **** | Health outcomes **** | Quality of care **** | Main results | Enablers / Barriers (for enrolment (E), utilization (U), impact (I) |
| **Female headed household** | | | | | | | | | | | | | | |
| *Africa* | | | | | | | | | | | | | | |
| Bamenda Ecclesiastical Provincial Health Assitance (BEPHA) (CBHI) | Cameroon | (Oraro et al. 2018) | Female headed households (based on household composition) | Obs | Survey among 550 households in north-west of Cameroon, with 2^nd^ highest level of health spending | Medium 9/11 points, 82% | Male headed households | + ^ | -^ |  |  |  | Higher percentage of female headed households is insured compared to male. Less outpatient and inpatient visits compared to male headed households, not significant. | Barrier (E): low SES, increased age, low trust in insurance, Enabling (E): children <15 years, church membership |
| CBHI | Ethiopia | (Mebratie et al. 2015) | Female headed households (not indicated how defined) | Obs Longitudinal household survey | 1,224 households in 2011, 1,203 in 2012, 87% is male headed household. | Medium 8/11 points  73% | Male headed households | 0 |  |  |  |  | Female headed households are less insured than male headed households (in descriptive data), though probability of enrolment for these groups shows no significant effect | Enabling (E): premium low compared to user fee, pressure from CBHI officials  Barriers: registration fee not affordable lack of awareness about scheme. |
| SHI, PHI, CBHI, Pre-payment scheme or other (mixed) | Kenya | (Kazungu & Barasa 2017) | Female headed households (not indicated how defined) | Obs, cross sectional | Secondary data from Kenya Demographic Health survey in 2009 and 2014, highest age in sample is 49 years. | Medium 11/14  79% | Male headed households | 0 |  |  |  |  | Men headed households had no great odds of being insured compared to female headed households. |  |
| CBHI (Mutual de Santé) | Rwanda | (Finnoff 2016) | Female headed household (not indicated how defined) | Obs, cross sectional | EICV2 (see reference in article), employed a two-stage stratified sample design, with a national sample of 34,785 individuals in 6,900 households. | Medium 9/11 points 82% | Male headed households | - | 0 |  |  |  | Female headed household were significantly less likely to participate in CBHI compared to male headed households.  Gender had no significance in determining use of health care service. |  |
| Community health fund (CBHI) | Tanzania | (Macha et al. 2014) | Female headed household (not indicated how defined) | Obs, cross sectional | 4 rural districts, 1,225 households (524 members, 701 non-members). 83% male headed households, mean age head: 44.4 | Medium 10/11 91% (Cross sectional) | Male headed households | - |  |  |  |  | CHF member household heads were less likely to be female than head of uninsured household heads. |  |
| *Asia* | | | | | | | | | | | | | | |
| PHI, SHI, CBHI (mixed) | India | (Chakrabarti & Shankar 2015) | Female headed households (head has decision making position) | Obs, empirical analysis in cross sectional study | Data from national family health survey 2005-2006, with 52,669 observations in rural areas and 40,591 observations in urban sample in 29 states in India. | Medium 10/11 points  91% | Male headed households | 0 |  |  |  |  | Households having female headed households did not have a higher enrolment pattern. For all health insurance schemes. CBHI is not effective in attracting households with a more dependent population. | Enabling (E): Living in urban area: odds for enrolling in PHI or public HI in comparison to no HI are higher. |
| Scheme (SHI, PHI, CBHI, mixed) | Country | Reference | Vulnerable group included | Study type ** | Study context, recruitment and sample | Quality grade  (CASP)*** | Comparison group | Enrolment **** | Utilization **** | Financial protection **** | Health outcomes **** | Quality of care **** | Main results | Enablers / Barriers (for enrolment (E), utilization (U), impact (I) |
| **Ethnic minorities** | | | | | | | | | | | | | | |
| *Asia* | | | | | | | | | | | | | | |
| Community health insurance (CBHI) | India | (Devadasan et al. 2010) | Ethnic minority (Adivasis)  with any ailment lasting longer than 30 days | QEx | After matching and snowball technique households were identified: 297 insured Adivasi households and 248 uninsured. Weekly follow up for 12 months. | High 11/12 points,  92% | Non-insured | + | + |  |  |  | Insured had higher incidence of chronic and major ailment.  Insured patients had 2.2 higher hospital admission rate than noninsured.  Of the Adivasis population, vulnerable sections of the insured populations (children, pregnant women and the poorest) had the highest hospital admission rates. | Enabling factor: trust in scheme |
| National scheme (SHI) | Thailand | (Hu 2010) | Ethnic minorities | Obs, cross sectional | Thai ethnic minority, n=198 (’00) and n=71 (’04), The Kanchanaburi Demographic Surveillance System (KDSS) | Medium 9/11 points, 82% | Thai citizens |  | 0 |  |  |  | Ethnic minority migrants had the lowest HI coverage and lowest level of health care service use. Health insurance increased by 26.5% (sign) between ’00 and ’04. The higher the HI, the higher level of service use. |  |
| Social health insurance (SHI) | Vietnam | (Palmer 2014) | Ethnic minorities, individuals reporting an ethnic- ity, other than Kinh or Chinese, from 54 ethnic groups | Obs, cross-sectional | Household and community information Survey (VHLSS), n= 6.188, with co-variate matching between insured and uninsured. | Medium 10/11 points, 91% | Other groups, based on socio demographics, e.g. persons with disabilities |  | + | + |  |  | For individuals reporting an ethnicity other than Kinh or Chinese, being insured increased the likelihood to use inpatient care (length of stay in hospital), insured had higher community clinic usage and insured had lower CHE. Insurance decrease probability of self-treatments. |  |
| Scheme (SHI, PHI, CBHI, mixed) | Country | Reference | Vulnerable group included | Study type ** | Study context, recruitment and sample | Quality grade  (CASP)*** | Comparison group | Enrolment **** | Utilization **** | Financial protection **** | Health outcomes **** | Quality of care **** | Main results | Enablers / Barriers (for enrolment (E), utilization (U), impact (I) |
| Displaced populations | | | | | | | | | | | | | | |
| *Asia* | | | | | | | | | | | | | | |
| National scheme (SHI) | Thailand | (Hu 2010) | Displaced populations, ethnic Chinese, Mon, Burmese/Twai, Karen, Khmer, Thai Yai, Yao, Karang, Nepalese, Yuan, or Vietnam | Obs | Thai ethnic minority, n=244 (’00) and n=100 (’04), The Kanchanaburi Demographic Surveillance System (KDSS) | Medium 9/11 points, 82% | Thai citizens | - | - |  |  |  | Ethnic minority migrants had the lowest HI coverage and lowest level of health care service use. Health insurance increased by 26.5% (sign) between ’00 and ’04. The higher the HI, the higher level of service use. |  |
| Thai Health insurance for people with citizenship problems (HI PCP) (SHI) | Thailand | (Suphanchaimat et al. 2016) | Displaced populations defined as stateless if he/she had a 13-digit national ID starting with 0 | Obs | Data source in patient records of one public hospital in 2009, 2011 and 2012. N=259, 66.2% female. | Medium 9/11 | Non-insured |  | + ^ |  |  |  | Membership of the stateless insurance with an increase in total admission per year compared to non-insured. | Enabling on utilization: for general population: older age, proximity to hospital, presence of catastrophic illness. |

References:

Aji B, Yamamoto SS, Sauerborn R. 2014. The economic impact of the insured patients with severe chronic and acute illnesses: A qualitative approach. *Global Health Action*, **7**.

Bernabe-Ortiz A, Diez-Canseco F, Vásquez A, Miranda JJ. 2016. Disability, caregivers dependency and patterns of access to rehabilitation care: Results from a national representative study in Peru. *Disability and Rehabilitation*, **38**: 582-588.

Bitran R, Escobar L, Gassibe P. 2010. After Chile's health reform: increase in coverage and access, decline in hospitalization and death rates. *Health Aff (Millwood)*, **29**: 2161-70.

Blay SL, Fillenbaum GG, Andreoli SB, Gastal FL. 2008. Equity of access to outpatient care and hospitalization among older community residents in Brazil. *Medical Care*, **46**: 930-937.

Castillo-Laborde C, Aguilera-Sanhueza X, Hirmas-Adauy M, et al. 2017. Health insurance scheme performance and effects on health and health inequalities in Chile. *MEDICC Review*, **19**: 57-64.

Chakrabarti A, Shankar A. 2015. Determinants of Health Insurance Penetration in India: An Empirical Analysis. *Oxford Development Studies*, **43**: 379-401.

Che YH, Chongsuvivatwong V, Li L, et al. 2016. Financial burden on the families of patients with hepatitis B virus-related liver diseases and the role of public health insurance in Yunnan province of China. *Public Health*, **130**: 13-20.

Cheng L, Liu H, Zhang Y, Shen K, Zeng Y. 2015. The impact of health insurance on health outcomes and spending of the elderly: Evidence from china's new cooperative medical scheme. *Health Economics (United Kingdom)*, **24**: 672-691.

Christiani Y, Byles JE, Tavener M, Dugdale P. 2017. Health insurance coverage among women in Indonesia's major cities: A multilevel analysis. *Health Care Women Int*, **38**: 267-282.

Daher H, Flessa S. 2010. Microfinance as a tool for financing medical devices in Syria. An assessment of needs and a call for further research. *Journal of Public Health*, **18**: 189-197.

de Menil VP, Knapp M, McDaid D, Njenga FG. 2014. Service use, charge, and access to mental healthcare in a private Kenyan inpatient setting: the effects of insurance. *PLoS One*, **9**: e90297.

Devadasan N, Criel B, Van Damme W, et al. 2010. Community health insurance in Gudalur, India, increases access to hospital care. *Health Policy Plan*, **25**: 145-54.

Doubova SV, Perez-Cuevas R, Canning D, Reich MR. 2015. Access to healthcare and financial risk protection for older adults in Mexico: secondary data analysis of a national survey. *BMJ Open*, **5**: e007877.

Dror DM, Soriano ES, Lorenzo ME, et al. 2005. Field based evidence of enhanced healthcare utilization among persons insured by micro health insurance units in Philippines. *Health Policy*, **73**: 263-71.

Duku SK, van Dullemen CE, Fenenga C. 2015. Does Health Insurance Premium Exemption Policy for Older People Increase Access to Health Care? Evidence from Ghana. *J Aging Soc Policy*, **27**: 331-47.

El-Sayed AM, Palma A, Freedman LP, Kruk ME. 2015. Does health insurance mitigate inequities in non-communicable disease treatment? Evidence from 48 low- and middle-income countries. *Health Policy*, **119**: 1164-75.

Fenny AP. 2017. Live to 70 Years and Older or Suffer in Silence: Understanding Health Insurance Status Among the Elderly Under the NHIS in Ghana. *J Aging Soc Policy*, **29**: 352-370.

Finnoff K. 2016. Gender Disparity in Access to the Rwandan Mutual Health Insurance Scheme. *Feminist Economics*, **22**: 26-50.

Goeppel C, Frenz P, Grabenhenrich L, Keil T, Tinnemann P. 2016. Assessment of universal health coverage for adults aged 50 years or older with chronic illness in six middle-income countries. *Bull World Health Organ*, **94**: 276-85C.

Gu L, Feng H, Jin J. 2017. Effects of Medical Insurance on the Health Status and Life Satisfaction of the Elderly. *Iran J Public Health*, **46**: 1193-1203.

Hu J. 2010. The role of health insurance in improving health services use by Thais and ethnic minority migrants. *Asia Pac J Public Health*, **22**: 42-50.

Huang J, Pan XL, Li A. 2013. Multi-level modelling of the factors that influence the participation of disabled rural individuals in social medical insurance in China. *BMC Health Serv Res*, **13**: 58.

Jiang C, Ma J, Zhang X, Luo W. 2012. Measuring financial protection for health in families with chronic conditions in Rural China. *BMC Public Health*, **12**: 988.

Jin Y, Hou Z, Zhang D. 2016. Determinants of Health Insurance Coverage among People Aged 45 and over in China: Who Buys Public, Private and Multiple Insurance. *PLoS One*, **11**: e0161774.

Jing S, Yin A, Shi L, Liu J. 2013. Whether New Cooperative Mmedical Schemes reduce the economic burden of chronic disease in rural China. *PLoS One*, **8**: e53062.

Kazungu JS, Barasa EW. 2017. Examining levels, distribution and correlates of health insurance coverage in Kenya. *Trop Med Int Health*, **22**: 1175-1185.

Lam KK, Johnston JM. 2012. Health insurance and healthcare utilisation for Shenzhen residents: a tale of registrants and migrants? *BMC Public Health*, **12**: 868.

Liao Y, Gilmour S, Shibuya K. 2016. Health Insurance Coverage and Hypertension Control in China: Results from the China Health and Nutrition Survey. *PLoS One*, **11**: e0152091.

Macha J, Kuwawenaruwa A, Makawia S, Mtei G, Borghi J. 2014. Determinants of community health fund membership in Tanzania: a mixed methods analysis. *BMC Health Serv Res*, **14**: 538.

Maurer J. 2008. Assessing horizontal equity in medication treatment among elderly Mexicans: which socioeconomic determinants matter most? *Health Econ*, **17**: 1153-69.

Mebratie AD, Sparrow R, Yilma Z, Alemu G, Bedi AS. 2015. Enrollment in Ethiopia’s Community-Based Health Insurance Scheme. *World Development*, **74**: 58-76.

Nguyen QLT, Van Phan T, Tran BX, et al. 2017. Health insurance for patients with HIV/AIDS in Vietnam: coverage and barriers. *BMC Health Serv Res*, **17**: 519.

Oraro T, Ngube N, Atohmbom GY, Srivastava S, Wyss K. 2018. The influence of gender and household headship on voluntary health insurance: the case of North-West Cameroon. *Health Policy Plan*, **33**: 163-170.

Palmer MG. 2014. Inequalities in universal health coverage: evidence from Vietnam. *World Development*, **64**: 384-394.

Palmer MG, Nguyen TMT. 2012. Mainstreaming health insurance for people with disabilities. *Journal of Asian Economics*, **23**: 600-613.

Parmar D, De Allegri M, Savadogo G, Sauerborn R. 2014a. Do community-based health insurance schemes fulfill the promise of equity? A study from Burkina Faso. *Health Policy Plan*, **29**: 76-84.

Parmar D, Williams G, Dkhimi F, et al. 2014b. Enrolment of older people in social health protection programs in West Africa--does social exclusion play a part? *Soc Sci Med*, **119**: 36-44.

Philip NE, Kannan S, Sarma SP. 2016. Utilization of Comprehensive Health Insurance Scheme, Kerala: A Comparative Study of Insured and Uninsured Below-Poverty-Line Households. *Asia Pac J Public Health*, **28**: 77S-85S.

Rivera-Hernandez M, Galarraga O. 2015. Type of Insurance and Use of Preventive Health Services Among Older Adults in Mexico. *J Aging Health*, **27**: 962-82.

Rivera-Hernandez M, Rahman M, Mor V, Galarraga O. 2016. The Impact of Social Health Insurance on Diabetes and Hypertension Process Indicators among Older Adults in Mexico. *Health Serv Res*, **51**: 1323-46.

Sun Q, Liu X, Meng Q, et al. 2009. Evaluating the financial protection of patients with chronic disease by health insurance in rural China. *Int J Equity Health*, **8**: 42.

Suphanchaimat R, Prakongsai P, Limwattananon S, Mills A. 2016. Impact of the health insurance scheme for stateless people on inpatient utilization in Kraburi Hospital, Thailand. *Risk Manag Healthc Policy*, **9**: 261-269.

Vialle-Valentin CE, Serumaga B, Wagner AK, Ross-Degnan D. 2015. Evidence on access to medicines for chronic diseases from household surveys in five low- and middle-income countries. *Health Policy Plan*, **30**: 1044-52.

Wang J, Chen L, Ye T, Zhang Z, Ma J. 2014. Financial protection effects of modification of China's New Cooperative Medical Scheme on rural households with chronic diseases. *BMC Health Serv Res*, **14**: 305.
