# Supplementary material for: Leaving no one behind? Social inclusion of health insurance in low- and middle-income countries: a systematic review
Source: Int J Equity Health. 2019 Aug 28;18:134. doi: 10.1186/s12939-019-1040-0 (PMC6714392; doi:10.1186/s12939-019-1040-0)
Supplement: Supplementary file 3 — Low quality studies. Low quality studies. (DOCX 37 kb) [file 12939_2019_1040_MOESM3_ESM.docx]

Additional file 3. Low quality studies

Study characteristics of excluded studies due to insufficient methodological quality

| Scheme (SHI, PHI, CBHI, mixed) | Country | Reference | Vulnerable group included | Study type ** | Study context, recruitment and sample | Main results |
| --- | --- | --- | --- | --- | --- | --- |
| SHI | Ghana | (1) | Aged | cross-sectional survey | Purposive sampling to group communities into urban rural. 2 urban cities. 10 rural communities. 210 respondents included. | Age of the people had no significant influence to the individual decision to enrol. |
| SHI | China | (2) | Aged with chronic conditions | Cross-cross sectional | Latest NCMS data 2009. Nationwide survey.  4454 NCMS participants. Mean age 46.2. 9 provinces. Ratio male to female 1.28. elders: 7.7% of sample (345/4454). Ratio male to female among elders 1.56. | Little attention to preventive part of care for rural elderly, low rates of treatment and use of preventive health care services NCMS partially reimburses medical expenses of chronic condition treatment. |
| SHI (Urban Employee-Basic Medical Insurance (UE-BMI) and Urban Residents-Basic Medical Insurance (UR-BMI) | China | (3) | Disability (schizophrenia, mental condition) | Cross-sectional | Comparing 2 schemes for treatment of schizophrenia, Field survey of policy documents, and hospitalization data of 2010 database of social insurance companies. Supplemented with in-depth interviews. | The experience of patients hospitalized with schizophrenia was significantly influenced by the insurance scheme that covered their illness. The data analysis stated that: firstly, the inpatients of UE-BMI utilized higher level medical institutions than inpatients of UR-BMI. Secondly, the inpatients of UE-BMI had higher proportion of patients receiving expensive drugs than those with UR-BMI coverage. Thirdly, the SGA prescription rate of in- patients of UE-BMI was significantly higher than those of UR-BMI. Since out-patient care was not integrated in UR-BMI scheme, members had 3 times longer average length of stay compared with those of UE-BMI. |
| SHI | Vietnam | (4) | Ethnic minorities | Cross sectional | Vhlss, 2006 and 208, Vietnam household living standards survey. | For ethnic groups between 2006-10 an increase of enrollment was found. especially in rural areas. |
| Mixed (SHI, CBHI, PHI) | India | (5) | Disability (psychiatric conditions) | Cross-sectional | Secondary macro data for 3 years. 2011-2012 and 2013 – 2014. Significant at 0.05. mandatory submission of data by the various entities that offer health insurance. | The penetration of health insurance is low and claim for mental illness remains low. The difference in patterns of age, gender, amount of claims, and number of days for mental illness provides detailed relevant information to formulate future policies |
| SHI | Thailand | (6) | Elderly | Cross sectional | 2 surveys 2003 and 2009, 8951 and 11402 older Thai, national representative surveys. | Pro rich inequality among elderly for oral care. |
| CBHI and PHI | India | (7) | Disabled  Chronically ill | Qualitative | From several practices or health care or insurance providers. Ethnographic interviews, several times, no comparison group, 63 community members hailing from a mix of social classes, nine public and 11 private practitioners as well as three healthcare administrators and 10 representatives of private and public insurance providers. | Barrier to health insurance: (mis)trust, misunderstanding of schemes, merits on perception of quality of care. |
| SHI | Ghana | (8) | Chronically ill | Qualitative | 32 individual interviewees, from the polyclinic, private clinics, pharmacies, and chemical and herbal shops. | Our preliminary analysis of diabetes experiences showed that few individuals (seven out of 20 respondents) had subscribed to the National Health Insurance Scheme. Poverty was the dominant reason provided for opting out of NHIS. For the minority who had NHIS subscription, challenges still existed with access to prescribed medications that were not on the NHIS exemption list |
| SHI | China | (9) | Disabled | Qualitative | Interviews and observations from visits to households with disabled members.  Six women, 14 men, aged 4-72, various education and living standards mainly poor and 5 average income and severity of impairment. Interviewed households rather than individuals. | To summarize, for the Wang family and other disabled households we interviewed in Harqin, the xinnonghe has had a positive but limited impact. Although it has reduced the Wang family’s medical expenses, the household still has to rely on loans to be able to access medical care in hospitals. The high out- of-pocket expenses for the healthcare services remain a major problem. It is the high costs that cause catastrophic health expenses and that ‘undermine the effect of any health insurance system in financial protection’ |
| SHI (Urban Employee-Basic Medical Insurance (UE-BMI) and Urban Residents-Basic Medical Insurance (UR-BMI) | China | (3) | Disability (schizophrenia, mental condition) | Cross-sectional | Comparing 2 schemes for treatment of schizophrenia, Field survey of policy documents, and hospitalization data of 2010 database of social insurance companies. Supplemented with in-depth interviews. | The experience of patients hospitalized with schizophrenia was significantly influenced by the insurance scheme that covered their illness. The data analysis stated that: firstly, the inpatients of UE-BMI utilized higher level medical institutions than inpatients of UR-BMI. Secondly, the inpatients of UE-BMI had higher proportion of patients receiving expensive drugs than those with UR-BMI coverage. Thirdly, the SGA prescription rate of in- patients of UE-BMI was significantly higher than those of UR-BMI. Since out-patient care was not integrated in UR-BMI scheme, members had 3 times longer average length of stay compared with those of UE-BMI. |

Quality assessment of excluded studies due to insufficient methodological quality.

| **Cross sectional studies** | | | | | | | | | | | |  |  |  |
| --- | --- | --- | --- | --- | --- | --- | --- | --- | --- | --- | --- | --- | --- | --- |
| Item number | 1 | 2 | 3 | 4 | 5 | 6 | 7 | 8 | 9 | 10 | 11 | Total score % | Total score | Potential bias |
| Topic of each item CASP cross sectional studies: 1 = clearly focused issue; 2 = appropriate method to answer research question; 3 = recruitment cases; 4 = Non-applicable; 5 exposure measured accurately; 6A = confounding factors accounted for; 6B = confounding factors in design/analysis; 7 = clarity of results; 8 = precision of results; 9 = reliability results; 10 = application to local situation; 11 = extend to what results fit with other available evidence. | | | | | | | | | | | | | | |
| Amo (1) | Y | Y | Y | NA | N | 6A Y  6B N | N | N | Y | N | N | 45 | 5/11 | Unclear measurement of insured vs noninsured, no sub-group analysis, results are not interpretable for vulnerable group, no transfer to local situation and other available evidence. |
| Dai 2015 (2) | Y | Y | Y | NA | Y | 6A N  6B N | Y | N | Y | N | Y | 64 | 7/11 | Few confounders, subgroup not in analysis, small subgroup populations, no generalization possible. |
| Feng (3) | Y | Y | CT | NA | Y | 6A N  6B N | Y | N | Y | N | N | 55 | 6/11 | No information about recruitment, use of hospital data, no confounders counted for and in analysis, no info on significant rate, no comparison with other results. |
| Long (4) | Y | N | N | NA | N | 6A N  6B N | Y | N | N | CT | Y | 27 | 3/11 | No defined aim, no clear recruitment, subjective data, insufficient measurement of outcome, no confounding factors, no statistics including p values, hard to assess reliability of results, no transfer to local situation. |
| Mohandoss (5) | CT | Y | Y | NA | N | 6A N  6B N | Y | Y | Y | Y | N | 55 | 6/11 | No clearly focused issue, no definition of target population, data collection and analysis not sufficiently explained, other than saying that data are deficient. |
| Somkortra (6) | Y | Y | CT | NA | N | 6A N  6B N | N | Y | Y | Y | Y | 55 | 6/11 | No information on recruitment, on outcome inadequate outcome measures (only use and type of service), no confounders taken into account, lack of clarity of results. |

| **Qualitative** | **1** | **2** | **3** | **4** | **5** | **6** | **7** | **8** | **9** | **10** |  |  |  |  |
| --- | --- | --- | --- | --- | --- | --- | --- | --- | --- | --- | --- | --- | --- | --- |
| 1 = clearly focused issue; 2 = qualitative method appropriate; 3 = research design meets aims; 4 = recruitment strategy; 5 = data collected to address research issue; 6 = relationship researcher and participants considered; 7 = ethical issues considered; 8 = data analysis sufficiently rigorous; 9 = clear statement of results; 10 = how valuable is research. | | | | | | | | | | | | | | |
| Ahlin (7) | Y | Y | N | Y | N | N | N | N | Y | Y |  | 50 | 5/10 | No information about methodology and interview guide, no transparency about analysis, no consideration regarding researcher versus participant, data analysis insufficient in rigor. |
| Aikins (8) | Y | Y | CT | N | Y | N | N | N | N | Y |  | 40 | 4/10 | Aims not discussed, no mention of recruitment strategy or relationship between participants and researcher, no ethical issues considered, unclear statements of analysis and results. |
| Sagli (9) | Y | Y | N | NA | CT | CT | N | CT | N | Y |  | 30 | 3/10 | Lacking explanation of research methods, can’t tell how data collected, interview guide, no data on analysis, no ethical issues considered, no clear statements of results. |
| Feng (3) | N | N | NA | NA | NA | NA | NA | NA | NA | NA |  | 0 | 0/10 | Additional qualitative study: not matching the aim of study. |

References

1. Amo T. The National Health Insurance Scheme (NHIS) in the Dormaa Municipality, Ghana: why some residents remain uninsured? Global journal of health science. 2014;6(3):82-9.

2. Dai B. Does China's new cooperative medical scheme promote rural elders' access to healthcare services in relation to chronic conditions? Int Health. 2015;7(1):32-41.

3. Feng Y, Xiong X, Xue Q, Yao L, Luo F, Xiang L. The impact of medical insurance policies on the hospitalization services utilization of people with schizophrenia: A case study in Changsha, China. Pak J Med Sci. 2013;29(3):793-8.

4. Long GT. Delivering Social Protection to the Poor and Vulnerable groups in Vietnam, Challenges and the role of the Government. ASEAN Economic Bulletin. 2012;29(3):245-58.

5. Mohandoss AA, Thavarajah R. An Audit of Indian Health Insurance Claims for Mental Illness from Pooled Insurance Information Bureau's Macroindicator Data. Indian J Psychol Med. 2017;39(3):254-61.

6. Somkotra T. Inequality in oral health-care utilisation exists among older Thais despite a universal coverage policy. Australas J Ageing. 2013;32(2):110-4.

7. Ahlin T, Nichter M, Pillai G. Health insurance in India: What do we know and why is ethnographic research needed. Anthropology and Medicine. 2016;23(1):102-24.

8. De-Graft Aikins A, Kushitor M, Koram K, Gyamfi S, Ogedegbe G. Chronic non-communicable diseases and the challenge of universal health coverage: Insights from community-based cardiovascular disease research in urban poor communities in Accra, Ghana. BMC Public Health. 2014;14(SUPPL. 2).

9. Sagli G, Zhang J, Ingstad B, Fjeld HE. Poverty and disabled households in the People’s Republic of China: experiences with a new rural health insurance scheme. Disability & Society. 2013;28(2):218-31,.
