# Supplementary material for: Leaving no one behind? Social inclusion of health insurance in low- and middle-income countries: a systematic review
Source: Int J Equity Health. 2019 Aug 28;18:134. doi: 10.1186/s12939-019-1040-0 (PMC6714392; doi:10.1186/s12939-019-1040-0)
Supplement: Supplementary file 4 — Quality assessment. Quality assessment based on forms from CASP. (DOCX 73 kb) [file 12939_2019_1040_MOESM4_ESM.docx]

Additional file 3

Quality assessment based on forms from CASP (CASP UK 2018)

Included studies

| **Cross sectional studies** | | | | | | | | | | | |  |  |  |
| --- | --- | --- | --- | --- | --- | --- | --- | --- | --- | --- | --- | --- | --- | --- |
| Item number | 1 | 2 | 3 | 4 | 5 | 6 | 7 | 8 | 9 | 10 | 11 | Total score % | Total score | Potential bias |
| Topic of each item CASP cross sectional studies: 1 = clearly focused issue; 2 = appropriate method to answer research question; 3 = recruitment cases; 4 = Non-applicable; 5 exposure measured accurately; 6A = confounding factors accounted for; 6B = confounding factors in design/analysis; 7 = clarity of results; 8 = precision of results; 9 = reliability results; 10 = application to local situation; 11 = extend to what results fit with other available evidence. | | | | | | | | | | | | | | |
| (Mebratie et al. 2015) | Y | Y | CT | NA | N | 6A Y  6B Y | Y | Y | Y | Y | N | 73 | 8/11 | Unclear definition of target group, self-report on health status |
| (Parmar, Williams, et al. 2014) | Y | Y | CT | NA | Y | 6A Y  6B Y | Y | Y | Y | Y | Y | 91 | 10/11 | Only household head recruited |
| (Finnoff 2016) | Y | Y | Y | NA | Y | 6A Y  6B N | Y | Y | Y | N | Y | 82 | 9/11 | Potential confounding factors not in analysis, not generalizable other populations |
| (Macha et al. 2014) | Y | Y | Y | NA | Y | 6A Y  6B Y | Y | Y | Y | N | Y | 91 | 10/11 | Small sample |
| (Che et al. 2016) | Y | Y | Y | NA | N | 6A Y  6B Y | Y | Y | Y | Y | Y | 91 | 10/11 | Recall bias of assessing financial burden of the last 12 months. |
| (Jiang et al. 2012) | Y | Y | Y | NA | Y | 6A Y  6B Y | Y | Y | Y | Y | N | 91 | 10/11 | Poor comparison with other studies |
| (Jing et al. 2013) | Y | Y | N | NA | N | 6A Y  6B N | Y | Y | Y | Y | N | 73 | 8/11 | Higher proportion of households with older adults in intervention group, possible recall bias on expenditure |
| (Sun et al. 2009) | Y | Y | N | NA | Y | 6A N  6B Y | Y | Y | Y | Y | CT | 73 | 8/11 | Recall bias, self-reported chronic disease, no comparison with other studies, few confounders such as distance to services |
| (Wang et al. 2014) | Y | Y | Y | NA | N | 6A Y  6B N | Y | Y | Y | Y | N | 73 | 8/11 | Recall bias on expenditure, confounders not in analyses, no comparison with other studies |
| (Jin, Hou, and Zhang 2016) | Y | Y | Y | NA | N | 6A Y  6B N | Y | Y | Y | N | Y | 73 | 8/11 | Recall bias on self-report, not applicable to other populations, no subgroup analysis per age group in large sample |
| (Lam and Johnston 2012) | Y | Y | N | NA | Y | 6a Y  6B Y | Y | Y | Y | N | N | 73 | 8/11 | Selection bias by telephone sampling, poor generalizability to other populations and not compared with other studies |
| (Philip, Kannan, and Sarma 2016) | Y | Y | CT | NA | Y | 6A Y  6B N | Y | N | Y | N | Y | 73 | 8/11 | No report about significance level used for interpretation of results, not transferable to other populations (one district), no information about sampling |
| (Daher and Flessa 2010) | Y | Y | Y | NA | N | 6A Y  6B N | Y | N | Y | Y | N | 73 | 8/11 | Reports the needs rather than the factual situation, no levels of significance reported, weak comparison with other studies |
| (Palmer 2014) | Y | Y | N | NA | Y | 6A Y  6B Y | Y | Y | Y | Y | Y | 91 | 10/11 | Persons with disabilities were significantly older, adverse selection, unclear how participants were recruited/selected |
| (Nguyen et al. 2017) | Y | Y | N | NA | Y | 6A Y  6B N | Y | Y | Y | N | Y | 73 | 8/11 | Convenience sampling approach, no subgroup analysis |
| (Bitran, Escobar, and Gassibe 2010) | Y | Y | Y | NA | N | 6A Y  6B N | Y | N | Y | Y | Y | 73 | 8/11 | Method to assess outcome measure not presented, no comparison with non-insured in analysis, no information on significance value used |
| (Castillo-Laborde et al. 2017) | Y | Y | Y | NA | Y | 6A Y  6B N | Y | N | Y | Y | Y | 82 | 9/11 | Use of different information sources, no subgroup analysis |
| (Rivera-Hernandez et al. 2016) | Y | Y | CT | NA | Y | 6A N  6B Y | Y | Y | Y | Y | N | 73 | 8/11 | Recall bias, no confounders such as distance to health service, minimal comparison with other studies |
| (Rivera-Hernandez and Galarraga 2015) | Y | Y | CT | NA | Y | 6A N  6B Y | Y | Y | Y | Y | Y | 82 | 9/11 | No clear information on recruitment, poor consideration of confounders |
| (Maurer 2008) | Y | Y | CT | NA | Y | 6A Y  6B Y | Y | Y | Y | Y | N | 82 | 9/11 | No information provided on recruitment, minimal comparison with other studies |
| (Goeppel et al. 2016) | Y | Y | Y | NA | N | 6A Y  6B N | Y | Y | Y | Y | Y | 82 | 9/11 | Information about type of health insurance is lacking, confounders/ sub group analysis lacking in analyses. |
| (Vialle-Valentin et al. 2015) | Y | Y | Y | NA | N | 6A N  6B N | Y | Y | Y | Y | Y | 73 | 8/11 | No reported the type of insurance, no analyses linked to health insurance, lacking sub group analysis |
| (Duku, van Dullemen, and Fenenga 2015) | Y | Y | Y | NA | N | 6A Y  6B N | Y | Y | Y | Y | N | 73 | 8/11 | Limited info on questionnaire, no subgroup analysis or comparison insured vs non-insured, study results only compared with results in the same country where study conducted. |
| (Fenny 2017) | Y | Y | Y | NA | Y | 6A Y  6B N | Y | Y | Y | N | N | 73 | 8/11 | Very specific policies due to exemption rate for within older aged group (60-69 and 70+), no comparison with other studies. |
| (Gu, Feng, and Jin 2017) | Y | Y | Y | NA | Y | 6A Y  6B Y | Y | N | Y | Y | N | 82 | 9/11 | Large value to assess significance, no comparison with other studies or discussion about context and interpretation of results |
| (Chakrabarti and Shankar 2015) | Y | Y | Y | NA | Y | 6A Y  6B Y | Y | N | Y | Y | Y | 91 | 10/11 | P value not reported |
| (Christiani et al. 2017) | Y | Y | Y | NA | Y | 6A Y  6B Y | Y | Y | Y | Y | Y | 100 | 11/11 |  |
| (Blay et al. 2008) | Y | Y | Y | NA | Y | 6A Y  6B Y | Y | Y | N | Y | Y | 91 | 10/11 | Hard to believe results, population not representative because mostly descendants from European migrants. |
| (Huang, Pan, and Li 2013) | Y | Y | Y | NA | Y | 6A Y 6B N | Y | Y | Y | Y | Y | 91 | 10/11 | Comparison with data from other studies, not within study. No multivariate analysis. |
| (Bernabe-Ortiz et al. 2016) | Y | Y | Y | NA | Y | 6A Y  6B N | Y | N | Y | Y | Y | 82 | 9/11 | No sub group analysis, no value for significance given. |
| (Oraro et al. 2018) | Y | Y | N | NA | Y | 6A Y 6B Y | Y | Y | Y | N | Y | 82 | 9/11 | No application to the local population, small n on particular group of interest. |
| (Hu 2010) | Y | Y | Y | NA | Y | 6A Y 6B Y | Y | Y | Y | N | N | 82 | 9/11 | No application to the local population, poor comparison of findings compared with other studies. |
| (Suphanchaimat et al. 2016) | Y | Y | CT | NA | Y | 6A Y  6B N | Y | Y | Y | N | Y | 82 | 9/11 | No application to the local population, secondary data thus lacking information about recruitment and selection of sample. |
| **Case control** | **1** | **2** | **3** | **4** | **5** | **6a 6b** | **7** | **8** | **9** | **10** | **11** | **Total score %** | **Total score** |  |
| 1 = clearly focused issue; 2 = appropriate method to answer research question; 3 = recruitment cases; 4 = selection of controls acceptable; 5 exposure measured accurately; 6A = confounding factors accounted for; 6B = confounding factors in design/analysis; 7 = clarity of results; 8 = precision of results; 9 = reliability results; 10 = application to local situation; 11 = extend to what results fit with other available evidence. | | | | | | | | | | | | | | |
| (Liao, Gilmour, and Shibuya 2016) | Y | Y | Y | Y | Y | 6A Y  6B N | Y | Y | Y | N | Y | 83 | 10/12 | Lacking information on measurement of BP, Regions excluded poorest and richest regions, confounders not taken for outcome of health insurance |
| (Dror et al. 2005) | Y | Y | N | Y | Y | 6A Y  6B N | Y | Y | Y | N | N | 67 | 8/12 | Not clear how chronic illness is measured, confounders not taken into consideration in subgroup analysis, low level of generalizability due to heterogeneity of micro insurances, minimal fit with other studies |
| (Palmer and Nguyen 2012) | Y | Y | Y | N | Y | 6A Y  6B Y | Y | Y | Y | Y | N | 83 | 10/12 | Unclear how controls were recruited/selected, non-fitting with other studies |
| (Doubova et al. 2015) | Y | Y | Y | Y | N | 6A Y  6B N | Y | Y | Y | Y | Y | 83 | 10/12 | Not clear how health insurance status was measured, no subgroup analysis |
| (El-Sayed et al. 2015) | Y | Y | N | Y | Y | 6A Y  6B N | Y | Y | Y | Y | Y | 83 | 10/12 | Nationwide surveys unclear recruitment, |
| (Cheng et al. 2015) | Y | Y | Y | Y | N | 6A Y  6B Y | Y | Y | Y | Y | N | 83 | 10/12 | No clear how health insurance (outcome) was assessed, no comparison of results with other studies |
| (Devadasan et al. 2010) | Y | Y | Y | Y | Y | 6A Y  6B Y | Y | Y | Y | N | Y | 92 | 11/12 | Applies to one ethnic minority, in one country, cannot be applied to local population. |
| **Cohort** | **1** | **2** | **3** | **4** | **5** | **6** | **7** | **8** | **9** | **10** | **11** | **12** | **Total score (%)** |  |
| 1 = clearly focused issue; 2 = recruitment cohort; 3 = exposure measured accurately; 4 = outcome accurately measured; 5A = confounding factors identified; 5B = confounding factors in design/analysis; 6A = follow up complete; 6B = follow up long enough; 7 = clarity of results; 8 = precision of results; 9 = reliability results; 10 = application to local situation; 11 = extend to what results fit with other available evidence; 12 implications for practice. | | | | | | | | | | | | | | |
| (Kazungu and Barasa 2017) | Y | Y | N | Y | 5A Y  5B Y | 6A Y  6B Y | Y | Y | Y | N | Y | N | 11/14 (79%) | Possible self-report bias, not applicable to other populations, no reported implications for practice |
| (Parmar, De Allegri, et al. 2014) | Y | Y | Y | CT | 5A Y 5B Y | 6A N 6B Y | Y | Y | Y | N | Y | N | 10/14 (71%) | Lacking information about utilization was assessed, high rate of dropouts, conducted only in one province, unclear implications for practice. |
| (de Menil et al. 2014) | Y | Y | N | Y | 5A Y 5B Y | 6A N 6B Y | Y | N | Y | Y | Y | Y | 11/14 (79%) | No use of confidence intervals, poor comparisons, recruited participants possibly not representative for population |
| **Qualitative** | **1** | **2** | **3** | **4** | **5** | **6** | **7** | **8** | **9** | **10** |  |  |  |  |
| 1 = clearly focused issue; 2 = qualitative method appropriate; 3 = research design meets aims; 4 = recruitment strategy; 5 = data collected to address research issue; 6 = relationship researcher and participants considered; 7 = ethical issues considered; 8 = data analysis sufficiently rigorous; 9 = clear statement of results; 10 = how valuable is research. | | | | | | | | | | | | | | |
| (Macha et al. 2014) | Y | Y | Y | Y | N | N | Y | Y | Y | Y |  | 80% | 8/10 | No information about interview guide, no consideration regarding researcher versus participant |
| (Aji, Yamamoto, and Sauerborn 2014) | Y | Y | Y | N | Y | N | Y | Y | Y | Y |  | 80% | 8/10 | Unclear how participants were selected, relationship between researcher and participants not addressed |

References

Aji, B., S. S. Yamamoto, and R. Sauerborn. 2014. "The economic impact of the insured patients with severe chronic and acute illnesses: A qualitative approach."  *Global Health Action* 7 (1). doi: 10.3402/gha.v7.22526.

Bernabe-Ortiz, A., F. Diez-Canseco, A. Vásquez, and J. J. Miranda. 2016. "Disability, caregivers dependency and patterns of access to rehabilitation care: Results from a national representative study in Peru."  *Disability and Rehabilitation* 38 (6):582-8. doi: 10.3109/09638288.2015.1051246.

Bitran, R., L. Escobar, and P. Gassibe. 2010. "After Chile's health reform: increase in coverage and access, decline in hospitalization and death rates."  *Health Aff (Millwood)* 29 (12):2161-70. doi: 10.1377/hlthaff.2010.0972.

Blay, S. L., G. G. Fillenbaum, S. B. Andreoli, and F. L. Gastal. 2008. "Equity of access to outpatient care and hospitalization among older community residents in Brazil."  *Medical Care* 46 (9):930-7. doi: 10.1097/MLR.0b013e318179254c.

CASP UK. 2018. "Casp checklists. Critical Appraisal Skills Programme (CASP) " CASP UK, Accessed July. <http://www.Casp-uk.Net>. .

Castillo-Laborde, C., X. Aguilera-Sanhueza, M. Hirmas-Adauy, I. Matute, I. Delgado-Becerra, M. Nájera-De Ferrari, A. Olea-Normandin, and C. González-Wiedmaier. 2017. "Health insurance scheme performance and effects on health and health inequalities in Chile."  *MEDICC Review* 19 (2-3):57-64.

Chakrabarti, A., and A. Shankar. 2015. "Determinants of Health Insurance Penetration in India: An Empirical Analysis."  *Oxford Development Studies* 43 (3):379-401.

Che, Y. H., V. Chongsuvivatwong, L. Li, H. Sriplung, Y. Y. Wang, J. You, S. J. Ma, et al. 2016. "Financial burden on the families of patients with hepatitis B virus-related liver diseases and the role of public health insurance in Yunnan province of China."  *Public Health* 130:13-20. doi: 10.1016/j.puhe.2015.03.015.

Cheng, L., H. Liu, Y. Zhang, K. Shen, and Y. Zeng. 2015. "The impact of health insurance on health outcomes and spending of the elderly: Evidence from china's new cooperative medical scheme."  *Health Economics (United Kingdom)* 24 (6):672-91. doi: 10.1002/hec.3053.

Christiani, Y., J. E. Byles, M. Tavener, and P. Dugdale. 2017. "Health insurance coverage among women in Indonesia's major cities: A multilevel analysis."  *Health Care Women Int* 38 (3):267-82. doi: 10.1080/07399332.2016.1253697.

Daher, H., and S. Flessa. 2010. "Microfinance as a tool for financing medical devices in Syria. An assessment of needs and a call for further research."  *Journal of Public Health* 18:189-97.

de Menil, V. P., M. Knapp, D. McDaid, and F. G. Njenga. 2014. "Service use, charge, and access to mental healthcare in a private Kenyan inpatient setting: the effects of insurance."  *PLoS One* 9 (3):e90297. doi: 10.1371/journal.pone.0090297.

Devadasan, N., B. Criel, W. Van Damme, S. Manoharan, P. S. Sarma, and P. Van der Stuyft. 2010. "Community health insurance in Gudalur, India, increases access to hospital care."  *Health Policy Plan* 25 (2):145-54. doi: 10.1093/heapol/czp044.

Doubova, S. V., R. Perez-Cuevas, D. Canning, and M. R. Reich. 2015. "Access to healthcare and financial risk protection for older adults in Mexico: secondary data analysis of a national survey."  *BMJ Open* 5 (7):e007877. doi: 10.1136/bmjopen-2015-007877.

Dror, D. M., E. S. Soriano, M. E. Lorenzo, J. N. Sarol, Jr., R. S. Azcuna, and R. Koren. 2005. "Field based evidence of enhanced healthcare utilization among persons insured by micro health insurance units in Philippines."  *Health Policy* 73 (3):263-71. doi: 10.1016/j.healthpol.2004.11.018.

Duku, S. K., C. E. van Dullemen, and C. Fenenga. 2015. "Does Health Insurance Premium Exemption Policy for Older People Increase Access to Health Care? Evidence from Ghana."  *J Aging Soc Policy* 27 (4):331-47. doi: 10.1080/08959420.2015.1056650.

El-Sayed, A. M., A. Palma, L. P. Freedman, and M. E. Kruk. 2015. "Does health insurance mitigate inequities in non-communicable disease treatment? Evidence from 48 low- and middle-income countries."  *Health Policy* 119 (9):1164-75. doi: 10.1016/j.healthpol.2015.07.006.

Fenny, A. P. 2017. "Live to 70 Years and Older or Suffer in Silence: Understanding Health Insurance Status Among the Elderly Under the NHIS in Ghana."  *J Aging Soc Policy* 29 (4):352-70. doi: 10.1080/08959420.2017.1328919.

Finnoff, K. 2016. "Gender Disparity in Access to the Rwandan Mutual Health Insurance Scheme."  *Feminist Economics* 22 (3):26-50.

Goeppel, C., P. Frenz, L. Grabenhenrich, T. Keil, and P. Tinnemann. 2016. "Assessment of universal health coverage for adults aged 50 years or older with chronic illness in six middle-income countries."  *Bull World Health Organ* 94 (4):276-85C. doi: 10.2471/BLT.15.163832.

Gu, L., H. Feng, and J. Jin. 2017. "Effects of Medical Insurance on the Health Status and Life Satisfaction of the Elderly."  *Iran J Public Health* 46 (9):1193-203.

Hu, J. 2010. "The role of health insurance in improving health services use by Thais and ethnic minority migrants."  *Asia Pac J Public Health* 22 (1):42-50. doi: 10.1177/1010539509351183.

Huang, J., X. L. Pan, and A. Li. 2013. "Multi-level modelling of the factors that influence the participation of disabled rural individuals in social medical insurance in China."  *BMC Health Serv Res* 13:58. doi: 10.1186/1472-6963-13-58.

Jiang, C., J. Ma, X. Zhang, and W. Luo. 2012. "Measuring financial protection for health in families with chronic conditions in Rural China."  *BMC Public Health* 12:988. doi: 10.1186/1471-2458-12-988.

Jin, Y., Z. Hou, and D. Zhang. 2016. "Determinants of Health Insurance Coverage among People Aged 45 and over in China: Who Buys Public, Private and Multiple Insurance."  *PLoS One* 11 (8):e0161774. doi: 10.1371/journal.pone.0161774.

Jing, S., A. Yin, L. Shi, and J. Liu. 2013. "Whether New Cooperative Medical Schemes reduce the economic burden of chronic disease in rural China."  *PLoS One* 8 (1):e53062. doi: 10.1371/journal.pone.0053062.

Kazungu, J. S., and E. W. Barasa. 2017. "Examining levels, distribution and correlates of health insurance coverage in Kenya."  *Trop Med Int Health* 22 (9):1175-85. doi: 10.1111/tmi.12912.

Lam, K. K., and J. M. Johnston. 2012. "Health insurance and healthcare utilisation for Shenzhen residents: a tale of registrants and migrants?"  *BMC Public Health* 12:868. doi: 10.1186/1471-2458-12-868.

Liao, Y., S. Gilmour, and K. Shibuya. 2016. "Health Insurance Coverage and Hypertension Control in China: Results from the China Health and Nutrition Survey."  *PLoS One* 11 (3):e0152091. doi: 10.1371/journal.pone.0152091.

Macha, J., A. Kuwawenaruwa, S. Makawia, G. Mtei, and J. Borghi. 2014. "Determinants of community health fund membership in Tanzania: a mixed methods analysis."  *BMC Health Serv Res* 14:538. doi: 10.1186/s12913-014-0538-9.

Maurer, J. 2008. "Assessing horizontal equity in medication treatment among elderly Mexicans: which socioeconomic determinants matter most?"  *Health Econ* 17 (10):1153-69. doi: 10.1002/hec.1313.

Mebratie, A. D., R. Sparrow, Z. Yilma, G. Alemu, and A. S. Bedi. 2015. "Enrollment in Ethiopia’s Community-Based Health Insurance Scheme."  *World Development* 74:58-76. doi: 10.1016/j.worlddev.2015.04.011.

Nguyen, Q. L. T., T. Van Phan, B. X. Tran, L. H. Nguyen, C. Ngo, H. T. T. Phan, and C. A. Latkin. 2017. "Health insurance for patients with HIV/AIDS in Vietnam: coverage and barriers."  *BMC Health Serv Res* 17 (1):519. doi: 10.1186/s12913-017-2464-0.

Oraro, T., N. Ngube, G. Y. Atohmbom, S. Srivastava, and K. Wyss. 2018. "The influence of gender and household headship on voluntary health insurance: the case of North-West Cameroon."  *Health Policy Plan* 33 (2):163-70. doi: 10.1093/heapol/czx152.

Palmer, M.G. 2014. "Inequalities in universal health coverage: evidence from Vietnam."  *World Development* 64:384-94. doi: <http://dx.doi.org/10.1016/j.worlddev.2014.06.008>.

Palmer, M.G., and T.M.T. Nguyen. 2012. "Mainstreaming health insurance for people with disabilities."  *Journal of Asian Economics* 23 (5):600-13.

Parmar, D., M. De Allegri, G. Savadogo, and R. Sauerborn. 2014. "Do community-based health insurance schemes fulfill the promise of equity? A study from Burkina Faso."  *Health Policy Plan* 29 (1):76-84. doi: 10.1093/heapol/czs136.

Parmar, D., G. Williams, F. Dkhimi, A. Ndiaye, F. A. Asante, D. K. Arhinful, and P. Mladovsky. 2014. "Enrolment of older people in social health protection programs in West Africa--does social exclusion play a part?"  *Soc Sci Med* 119:36-44. doi: 10.1016/j.socscimed.2014.08.011.

Philip, N. E., S. Kannan, and S. P. Sarma. 2016. "Utilization of Comprehensive Health Insurance Scheme, Kerala: A Comparative Study of Insured and Uninsured Below-Poverty-Line Households."  *Asia Pac J Public Health* 28 (1 Suppl):77S-85S. doi: 10.1177/1010539515602306.

Rivera-Hernandez, M., and O. Galarraga. 2015. "Type of Insurance and Use of Preventive Health Services Among Older Adults in Mexico."  *J Aging Health* 27 (6):962-82. doi: 10.1177/0898264315569457.

Rivera-Hernandez, M., M. Rahman, V. Mor, and O. Galarraga. 2016. "The Impact of Social Health Insurance on Diabetes and Hypertension Process Indicators among Older Adults in Mexico."  *Health Serv Res* 51 (4):1323-46. doi: 10.1111/1475-6773.12404.

Sun, Q., X. Liu, Q. Meng, S. Tang, B. Yu, and R. Tolhurst. 2009. "Evaluating the financial protection of patients with chronic disease by health insurance in rural China."  *Int J Equity Health* 8:42. doi: 10.1186/1475-9276-8-42.

Suphanchaimat, R., P. Prakongsai, S. Limwattananon, and A. Mills. 2016. "Impact of the health insurance scheme for stateless people on inpatient utilization in Kraburi Hospital, Thailand."  *Risk Manag Healthc Policy* 9:261-9. doi: 10.2147/RMHP.S117173.

Vialle-Valentin, C. E., B. Serumaga, A. K. Wagner, and D. Ross-Degnan. 2015. "Evidence on access to medicines for chronic diseases from household surveys in five low- and middle-income countries."  *Health Policy Plan* 30 (8):1044-52. doi: 10.1093/heapol/czu107.

Wang, J., L. Chen, T. Ye, Z. Zhang, and J. Ma. 2014. "Financial protection effects of modification of China's New Cooperative Medical Scheme on rural households with chronic diseases."  *BMC Health Serv Res* 14:305. doi: 10.1186/1472-6963-14-305.
